# Supplementary figures and images for: DENV NS1 and MMP-9 cooperate to induce vascular leakage by altering endothelial cell adhesion and tight junction
Source: PLoS Pathog. 2021 Jul 26;17(7):e1008603. doi: 10.1371/journal.ppat.1008603 (PMC8341711; doi:10.1371/journal.ppat.1008603)

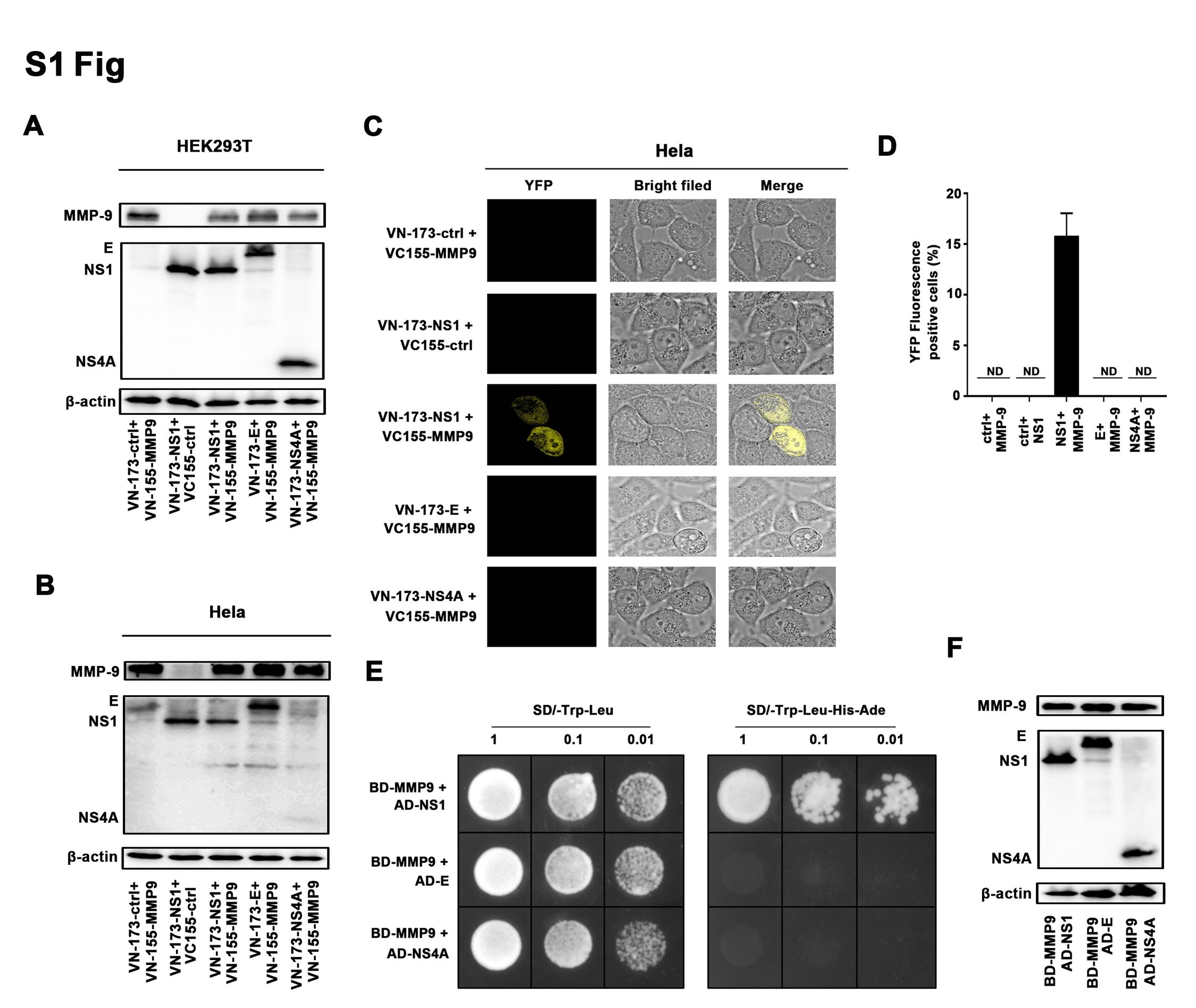

Supplement: S1 Fig — (A, B) HEK293T cells and Hela cells were was co-transfected with empty vector or VC-155-MMP-9 and VN173-NS1/E/NS4A. At 24 h post-transfection, cell lysates were analyzed by immunoblotting. (C, D) Hela cells were was co-transfected with empty vector or VC-155-MMP-9 and VN173-NS1/E/NS4A. At 24 h post-transfection, living cells were observed by confocal microscopy (C). The quantification of YFP-positive cells was used by ImageJ software (D). ND means not detected. (E, F) Yeast strain AH109 were co-transformed with combination of binding domain (BD-MMP-9) and activation domain (AD-NS1, AD-E, and AD-NS4A) plasmid. Transfected yeast cells were grown on SD-minus Trp/Leu double dropout plates, and colonies were replicated on to SD-minus Trp/Leu/Ade/His fourth dropout plates to check for the expression of reporter genes (E). Yeast strain AH109 were co-transformed with combination of binding domain (BD-MMP-9) and activation domain (AD-NS1, AD-E, and AD-NS4A) plasmid. At 48 h post-transfection, cell lysates were analyzed by immunoblotting (F). Dates were representative of two to three independent experiments. (TIF) [file ppat.1008603.s001.tif]

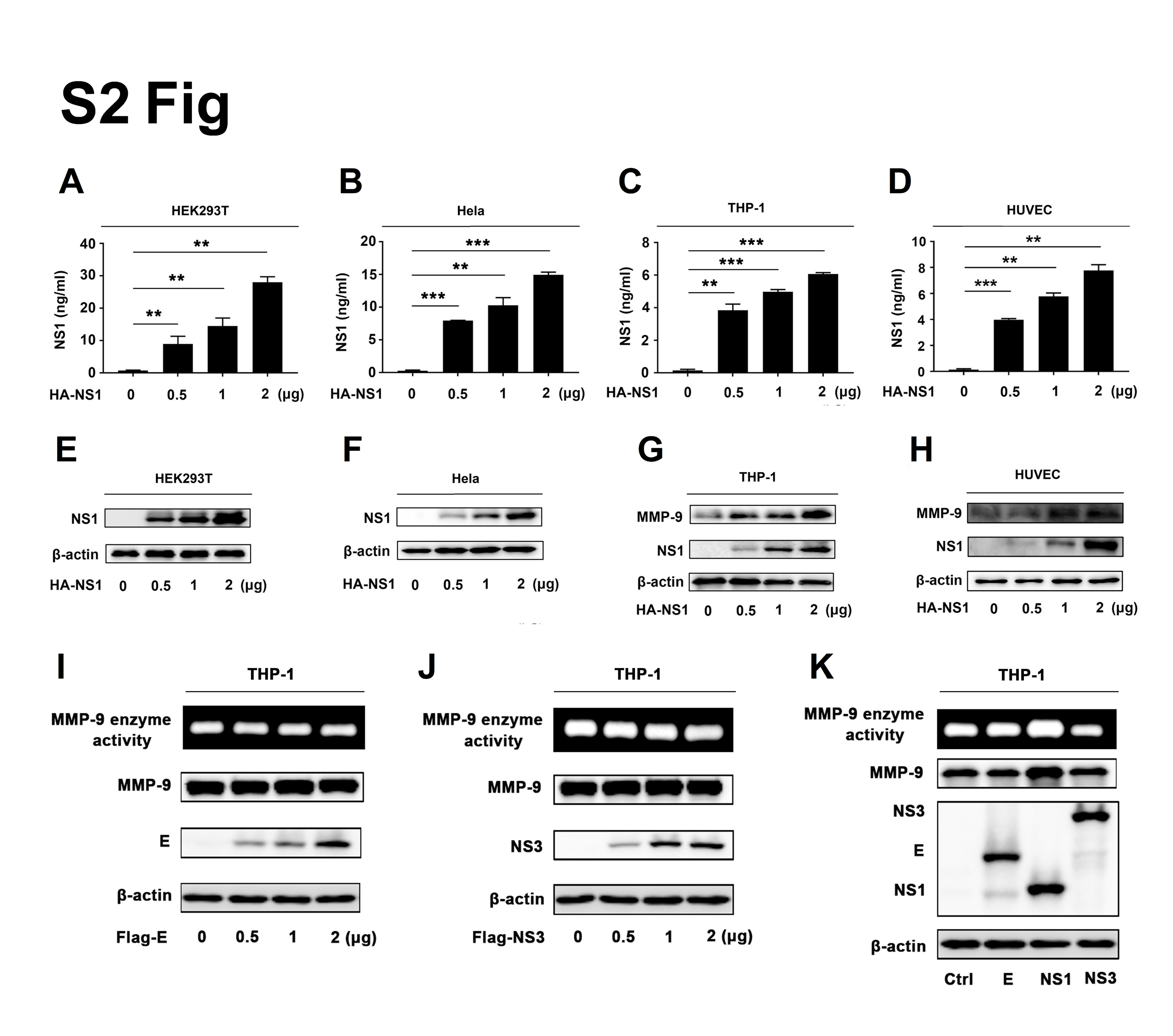

Supplement: S2 Fig — (A–H) HEK293T cell (A, E), Hela cells (B, F), PMA-differentiated THP-1 macrophages (C, G) and HUVECs (D, H) were transfected with the different concentrations of plasmid encoding NS1 for 24 h. NS1 protein in Supernatants were analyzed by ELISA (A–D). Cell lysates were analyzed (E–H) by immunoblotting. (I, J) PMA-differentiated THP-1 macrophages were transfected with the different concentrations of plasmid encoding E (I) or NS3 (J) for 24 h. Supernatants were analyzed (top) by gelatin zymography assays for MMP-9 proteinase activity. Cell lysates were analyzed (bottom) by immunoblotting. (K) PMA-differentiated THP-1 macrophages were transfected with the same concentrations of plasmid encoding NS1, E, or NS3 for 24 h. Supernatants were analyzed (top) by gelatin zymography assays for MMP-9 proteinase activity. Cell lysates were analyzed (bottom) by immunoblotting. Dates were representative of two to three independent experiments. Values are mean ± SEM, P ≤0.05 (*), P ≤0.01 (**), P ≤0.001 (***). (TIF) [file ppat.1008603.s002.tif]

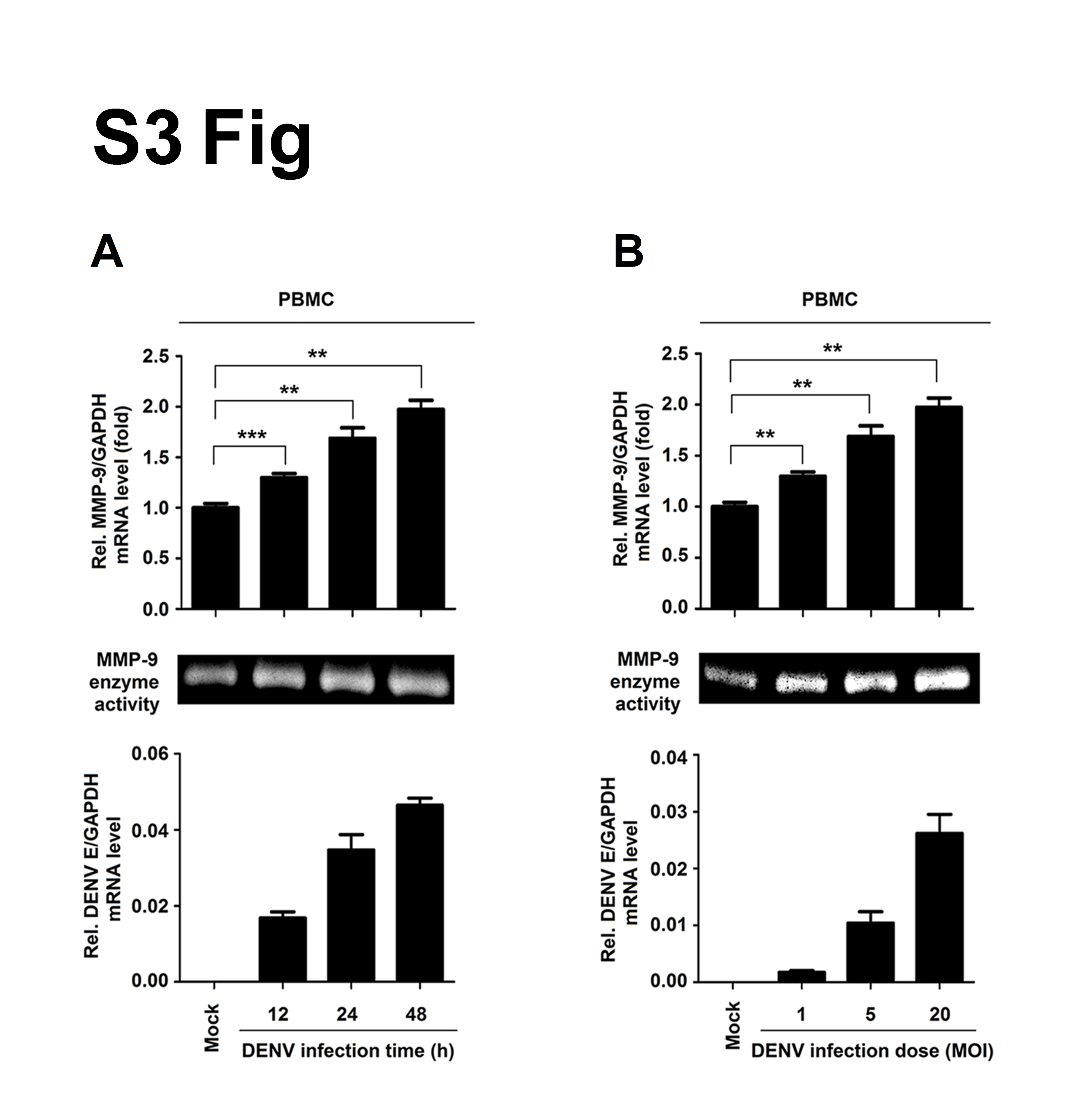

Supplement: S3 Fig — (A, B) Human PBMCs were infected with DENV2 for different times at MOI = 5 (A) or at different concentrations for 24 h (B). Intracellular MMP-9 RNA (top) and DENV2 E RNA (bottom) was determined by qRT-PCR analysis and MMP-9 proteinase activity in the supernatants was determined by gelatin zymography assays (middle). Dates were representative of two to three independent experiments. Values are mean ± SEM, P ≤0.05 (*), P ≤0.01 (**), P ≤0.001 (***). (TIF) [file ppat.1008603.s003.tif]

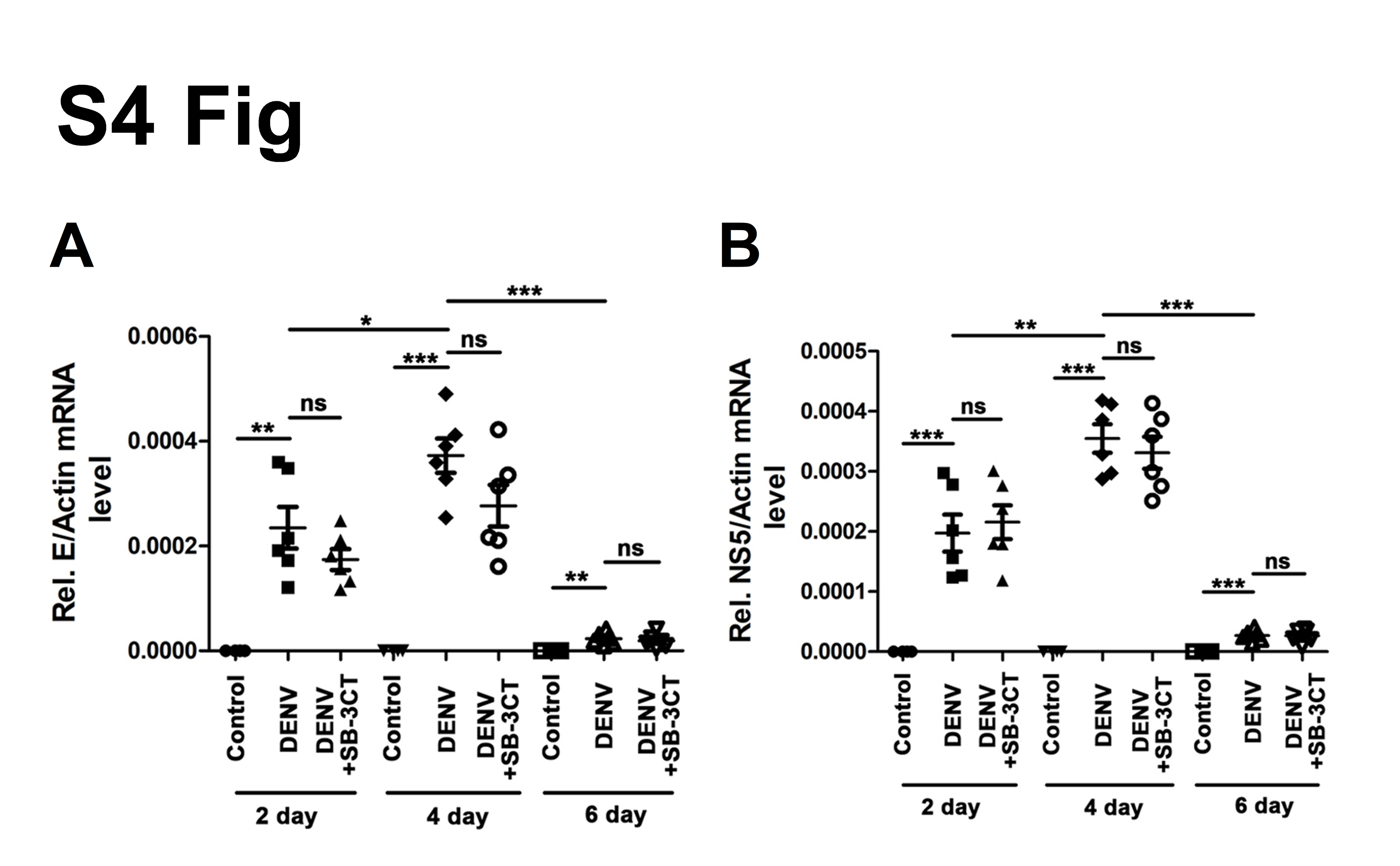

Supplement: S4 Fig — (A, B) IFNAR-/- C57BL/6 mice were intravenously injected with 300 μl DENV2 at a dose of 1×10^6^ PFU/mouse (n = 6), pre-treated with 300 μl PBS containing MMP-9 specific inhibitor SB-3CT (5 mg/kg per mice) by intraperitoneal injection for 90 min and then treated with DENV2 (1×10^6^ PFU/mouse), repeat treated with SB-3CT (5 mg/kg per mice) on the fourth day after DENV2 (NGC) infection (n = 6), or 300 μl PBS containing the same volume DMSO as a control group (n = 4). 7 days after infection, mice were euthanasia, and the tissues were collected. Blood samples were collected at 2, 4, and 6 days post-infection. DENV2 E (A) and NS5 (B) RNA was determined by qRT-PCR. Points represent the value of each blood samples. Dates were representative of two independent experiments. ns means not significant. P ≤0.05 (*), P ≤0.01 (**), P ≤0.001 (***). (TIF) [file ppat.1008603.s004.tif]

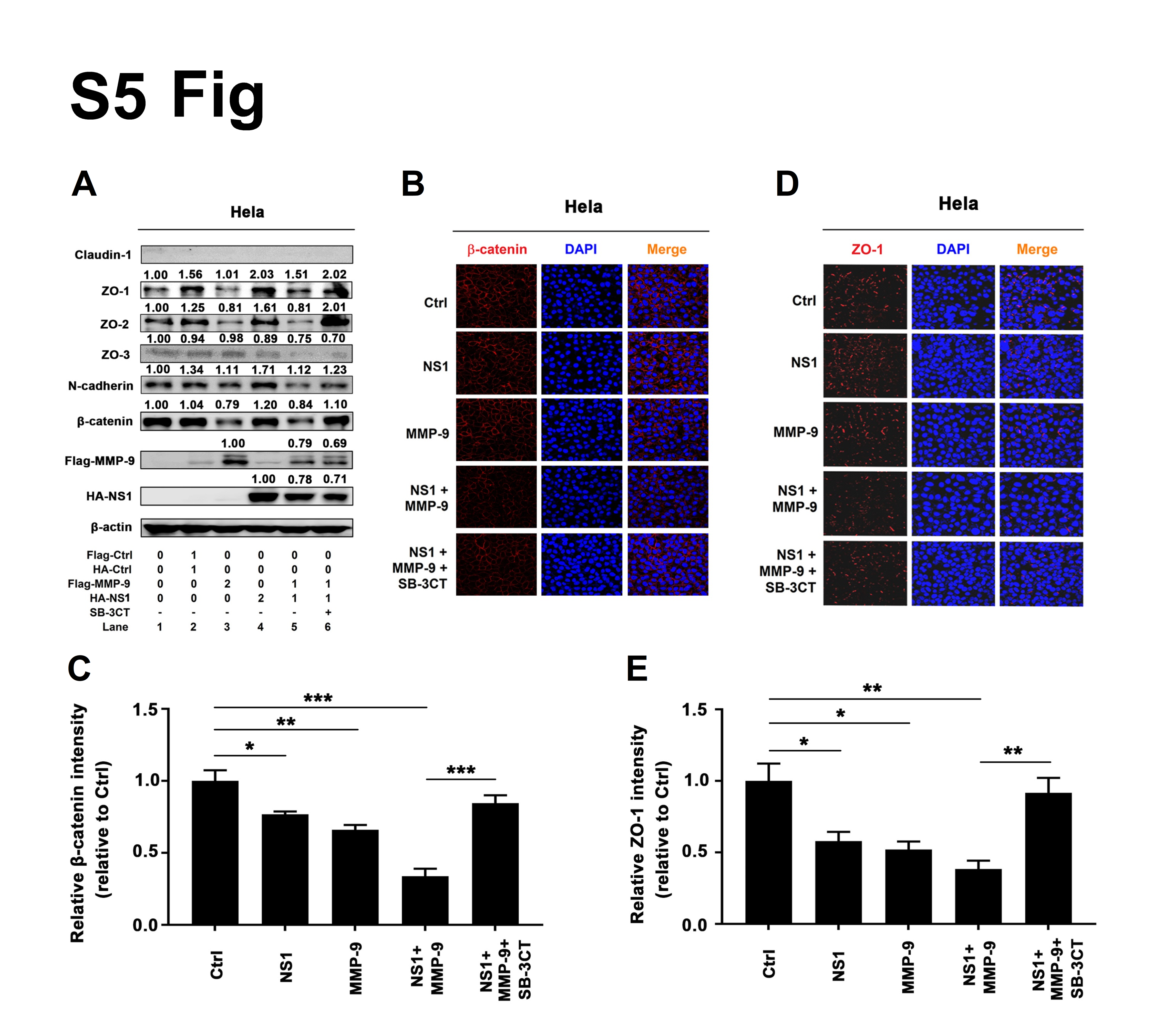

Supplement: S5 Fig — (A) Hela cells were respectively transfected with plasmid encoding MMP-9 (2 μg) or NS1 (2 μg) or NS1 (1 ug) plus MMP-9 (1 μg) for 24 h or firstly co-transfected with plasmid encoding NS1 (1ug) plus MMP-9 (1 μg) for 12 h, then treated with 600nM SB-3CT for 12 h. The indicated proteins in cell extract were analyzed by WB. (B–E) Hela cells were treated with NS1 protein (5 μg/ml) or MMP-9 protein (100 ng/ml) or NS1 (5 μg/ml) plus MMP-9 (100 ng/ml) or pre-incubated with 600nM SB-3CT for 1 h, then treated with NS1 (5 μg/ml) plus MMP-9 (100 ng/ml) for 6 h, The distribution of endogenous β-catenin (B) or ZO-1 (D) protein were visualized under confocal microscope. The quantification of relative β-catenin (C) or ZO-1 (E) intensity was used by ImageJ software. Dates were representative of three independent experiments. The quantification of protein was used by ImageJ software (A). (TIF) [file ppat.1008603.s005.tif]

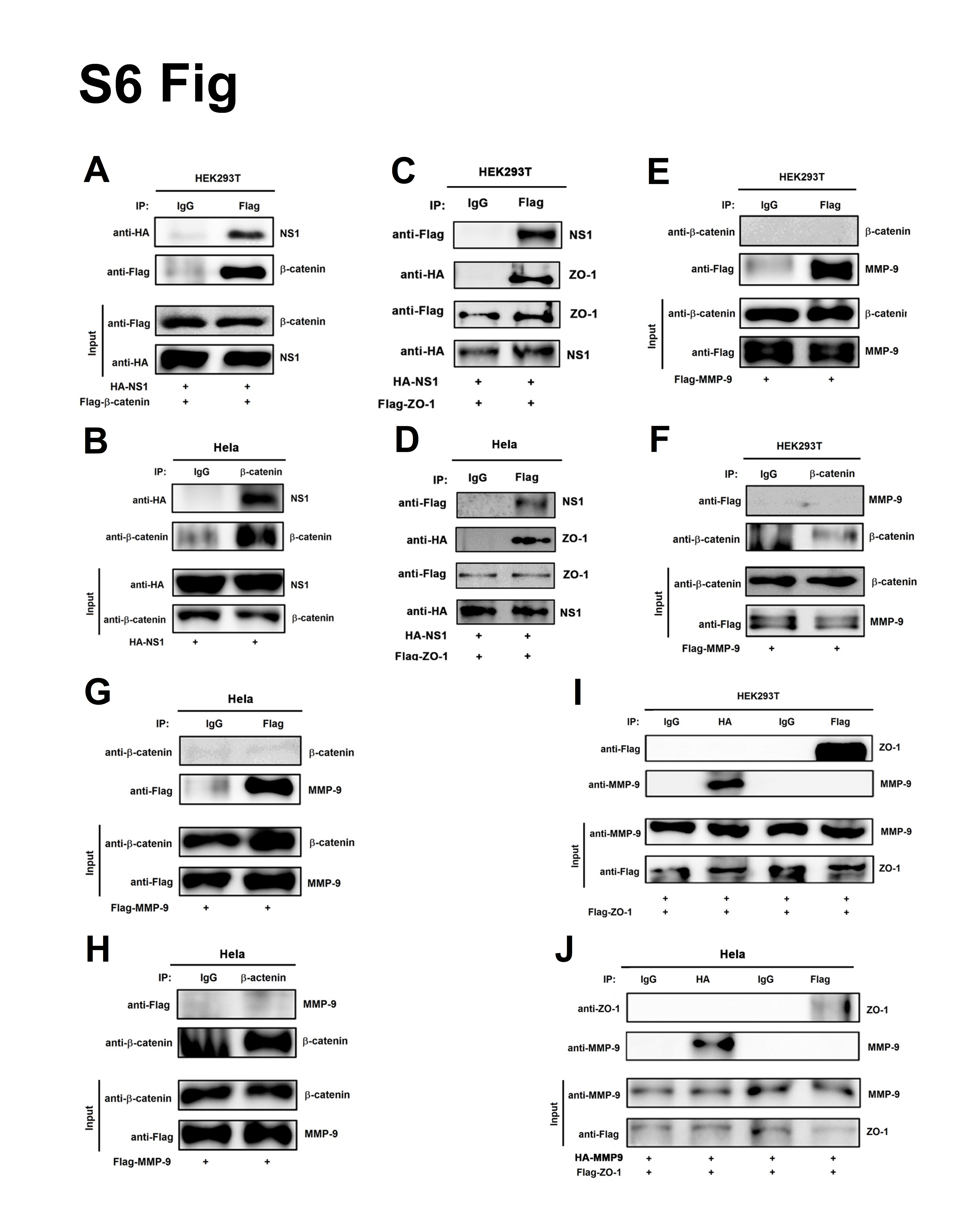

Supplement: S6 Fig — (A, C) HEK293T cells were transfected with plasmid encoding HA-NS1 plus Flag-β-catenin (A) or HA-NS1 plus Flag-ZO-1 (C). Cell lysates were immunoprecipitated using anti-Flag antibody, and analyzed using anti-Flag, anti-HA antibody. Cell lysates (40 μg) was used as Input. (B, D) Hela cells were transfected with plasmid encoding HA-NS1 (B) or HA-NS1plus Flag-ZO-1 (D). Cell lysates were immunoprecipitated using anti-β-catenin antibody (B) or anti-Flag (D), and analyzed using anti-Flag, anti-HA antibody and anti-β-catenin antibody. Cell lysates (40 μg) was used as Input. (E–H) HEK293T cells (E, F) or Hela cells (G, H) were transfected with plasmid encoding Flag-MMP-9. Cell lysates were immunoprecipitated using anti-Flag or anti-β-catenin antibody, and analyzed using anti-Flag or anti-β-catenin antibody. Cell lysates (40 μg) was used as Input. (I, J) HEK293T cells (I) or Hela cells (J) were transfected with plasmid encoding HA-MMP-9 plus Flag-ZO-1, Cell lysates were immunoprecipitated using anti-HA or anti-Flag antibody, and analyzed using anti-Flag or anti-MMP-9 antibody. Cell lysates (40 μg) was used as Input. All dates were representative of three independent experiments. (TIF) [file ppat.1008603.s006.tif]

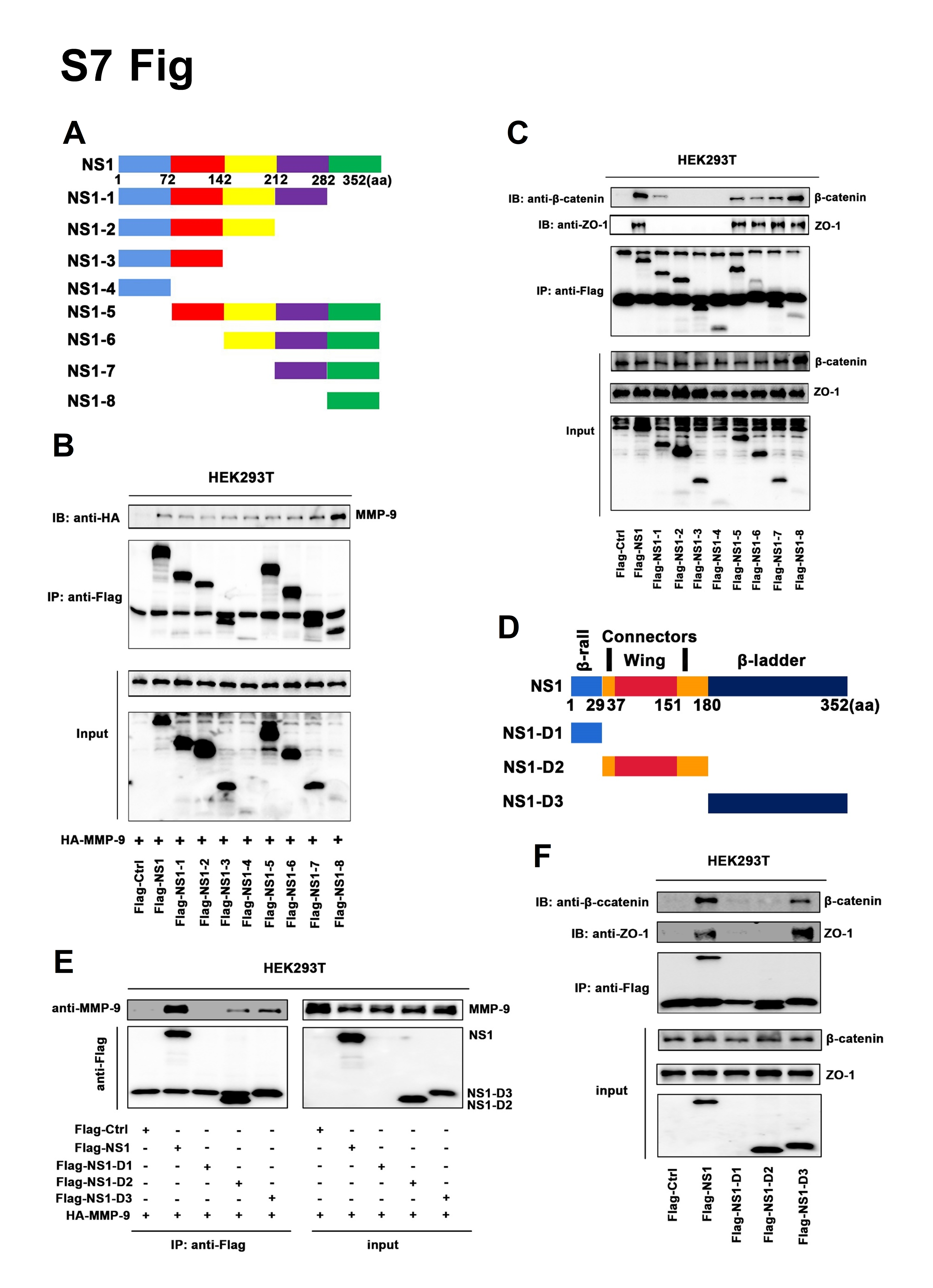

Supplement: S7 Fig — (A) Schematic diagram of wild-type NS1 protein and truncated mutants NS1 protein (NS1-1 to NS1-8). (B) HEK293T cells were co-transfected with HA-MMP-9 and Flag-NS1 and its truncated mutants (NS1-1 to NS1-2). Cell lysates were immunoprecipitataed using anti-Flag antibody, and analyzed using anti-Flag and anti-HA antibody. Cell lysates (40 μg) was used as Input. (C) HEK293T cells were transfected Flag-NS1 and its truncated mutants (NS1-1 to NS1-2). Cell lysates were immunoprecipitataed using anti-Flag antibody, and analyzed using anti-Flag, anti-b-catenin, and anti-ZO-1 antibodies. Cell lysates (40 μg) was used as Input. (D) Schematic diagram of wild-type NS1 protein and truncated different domains of NS1 protein (NS1-D1 to NS1-D3). (E) HEK293T cells were co-transfected with HA-MMP-9 and Flag-NS1 and its truncated domains (NS1-D1 to NS1-D3). Cell lysates were immunoprecipitataed using anti-Flag antibody, and analyzed using anti-Flag and anti-MMP-9 antibody. Cell lysates (40 μg) was used as Input. (F) HEK293T cells were transfected Flag-NS1 and its truncated domains (NS1-D1 to NS1-D3). Cell lysates were immunoprecipitataed using anti-Flag antibody, and analyzed using anti-Flag, anti-β-catenin, and anti-ZO-1 antibodies. Cell lysates (40 μg) was used as Input. All dates were representative of three independent experiments. (TIF) [file ppat.1008603.s007.tif]

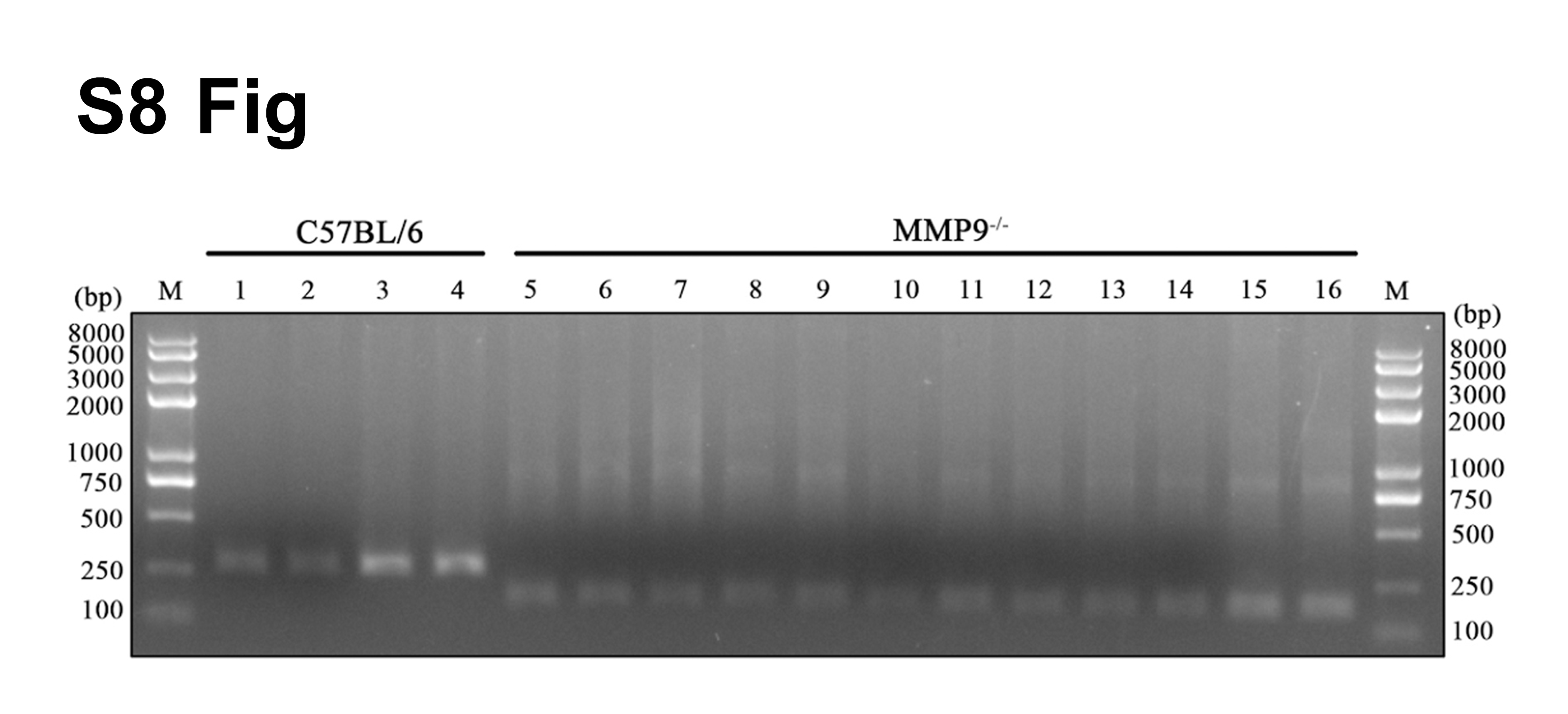

Supplement: S8 Fig — C57BL/6 mice and MMP-9-/- mice were injected intravenously DENV2 NS1 protein [10 mg/kg (n = 5)], the same volume of PBS was also tail vein injected to C57BL/6 mice and MMP-9-/- mice (n = 5) as control group. Another group of MMP-9-/- mice (n = 5) were injected intravenously DENV2 NS1 protein (10 mg/kg) plus recombinant mouse MMP-9 protein (70 μg/kg). After 24 h post-injection. The tails randomly selected from five groups (Four mice came from wild type C57BL/6 and twelve mice came from MMP-9-/- mice), the total genome was extracted from the tail of mice. The knock-out level of MMP-9 was detected by specific primers. (TIF) [file ppat.1008603.s008.tif]
