# Supplementary material for: DENV NS1 and MMP-9 cooperate to induce vascular leakage by altering endothelial cell adhesion and tight junction
Source: PLoS Pathog. 2021 Jul 26;17(7):e1008603. doi: 10.1371/journal.ppat.1008603 (PMC8341711; doi:10.1371/journal.ppat.1008603)
Supplement: Attachment — Submitted filename: PLoS Pathog Responses to the Reviews Pan-Wu 20201027.docx [file ppat.1008603.s013.docx]

**Responses to the Review Comments**

**Part I – Summary**

**Reviewer #1:** In this paper, Wu and colleagues report in vitro and in vivo studies on the effect of DENV NS1 protein and host protein matrix metalloproteinase-9 (MMP-9) on permeability of endothelial cells. An important function of DENV NS1 in decreasing endothelium permeability and severe dengue pathogenesis has been established. Previous reports indicate that DENV NS1 disrupts the endothelial glycocalyx layer to compromise the integrity of the vascular permeability barrier. It was also shown previously that DENV-infected dendritic cells trigger vascular leakage through MMP-9 overproduction. Here the authors link NS1 to MMP-9 overproduction and present the following arguments in this paper: 1) DENV NS1 transcriptionally increase MMP-9 expression via NFkB activation; 2) DENV NS1 inhibits TIMP-1 production; 3) DENV NS1 interacts with both MMP-9, ZO-1, and beta-catenin, recruiting MMP-9 to these junction proteins to facilitate the deterioration of both adhesion and tight junctions. Although the study provides strong data confirming the role of both NS1 and MMP-9 in DENV-induced vascular leakage, the mechanistic parts, which are the novel parts of the study, has drawbacks and appear preliminary at this stage.

**Authors’ Response:** Thank you for the summary.

**Reviewer #2:** Vascular leakage and hemorrhage are key features of severe dengue disease, so understanding the viral mechanisms that trigger endothelial permeability is important. Prior work has shown that the viral NS1 protein can promote endothelial permeability, but the mechanisms by which this occurs are not entirely clear. Here, Li and colleagues characterize a coordinated effect of NS1 and MMP9 on cell junction molecules and ultimately endothelial barrier permeability. The study is comprehensive, including patient samples, mouse models, and molecular studies. In general the experiments are thorough but additional clarifications would allow the authors to draw more robust conclusions.

**Authors’ Response:** Thank you for your summary and comments.

**Reviewer #2:** A weakness of the proposed mechanism is that the interaction experiments are all done in cells transfected with tagged NS1 and MMP9, rather than during infection; this may or may not represent the authentic localization of NS1 during infection, especially since NS1 is a secreted protein. The authors note cytoplasmic localization of NS1 in their transfection system (Fig 2, Line 171-172, Fig 9), but during infection NS1 is present in the ER lumen and secreted. Is NS1 secreted in this system? Do the authors propose that NS1 and MMP9 interact in the cytoplasm?

**Authors’ Response:** Thank you for the comments and for your good question.

(1) According to your suggestion, we carried out additional experiments to determine whether NS1 secrets in the cell systems in the revised manuscript.

HEK293T cells, Hela cells, THP-1 differentiated macrophages, and HUVECs were transfected with plasmid pHA-NS1 at different concentrations. ELISA results indicated that the levels of NS1 protein in the supernatants of HEK293T cells, Hela cells, THP-1 differentiated macrophages, and HUVECs were increased as the concentrations of NS1 increased (S1A–D Fig), demonstrating that NS1 protein is secreted in the supernatants of these cell systems.

(2) In addition, we have performed new experiments to confirm the interaction of NS1 with MMP9 in the revised manuscript. Notably, His-pull-down assays demonstrated that purified DENV2-NS1 protein could directly interact with purified MMP-9 protein, but purified SARS-CoV2-N protein failed to interact with purified MMP-9 protein (Revised Fig. 2C), indicating that DENV2 NS1 protein specifically and directly interact with MMP-9 protein.

**Reviewer #2:** Fig 1: If patient sera are still available, it would be helpful to show viremia by qRT-PCR. Is high NS1 per se important, or is high NS1 a marker for high viral load, which could impact vascular permeability by other mechanisms?

**Authors’ Response:** Thank you for the very good suggestion.

However, the patient sera are not available now, and therefore, we cannot do these tests.

**Reviewer #2:** Line 157: The correlation between TIMP1 and NS1 is not compelling.

**Authors’ Response:** Thank you for the comment.

According to your comment, we have performed additional experiments to determine the correlation between TIMP1 and NS1 in the revised manuscript.

HEK293T cells, Hela cells, and THP-1 differentiated macrophages were transfected with plasmid pHA-NS1 at different concentrations. We noted that the levels of TIMP-1 protein were reduced by NS1 in HEK293T cells, Hela cells, and THP-1 differentiated macrophages in dose-dependent manners (S1E–G Fig), indicating that DENV NS1 inhibits TIMP-1 production in these cells.

We have corrected the sentence from “However, there was a converse correlation between TIMP-1 and MMP-9 or NS1 (Fig 1E and F) in the sera of severe dengue patients.” to “However, there was an inverse correlation between TIMP-1 and MMP-9 or NS1 (Fig 1E and F) in the sera of severe dengue patients.” in the revised manuscript.

**Reviewer #2:** For qRT-PCR, it is not clear what expression levels are relative to (if they are already normalized to GAPDH). (Fig 3, Fig 4)

**Authors’ Response:** Thank you again.

In Fig. 3 and Fig. 4, we have used a relatively quantitative method to describe the expression mRNA levels. Each sample was the target gene relative to reference genes. In Fig. 4A, B and Revised S2A, B Fig: the values of expression levels were relative to normalized GAPDH. In Fig. 4C, D: the values were not normalized to GAPDH, and we have corrected the incorrectly marker the Y-axis in the revised manuscript (Revised Fig. 4C, D).

**Reviewer #2:** Fig 4E: Why is there a single mock for both cell types? If relative to GAPDH, these shouldn't be averaged together. It would be better to show viral loads based on a standard curve, on a Log10 scale, rather than relative to GAPDH.

**Authors’ Response:** Thank you for the comment.

Since viral RNA production was not detected in THP-1 cells or HUVECs, therefore, we have only used a single mock control. As you suggested, we have used the Log10 value instead to show the viral loads based on a standard curve in the revised manuscript (Revised Fig. 4E).

**Reviewer #2:** Fig 5B: Is it surprising that the MMP9 inhibitor reduces MMP9 levels? Does that suggest that this drug acts by blocking MMP9 production, rather than inhibiting its activity?

**Authors’ Response:** Thanks for the question.

We noted that MMP9 inhibitor SB-3CT had no effect on the level of MMP-9 RNA (Revised Fig. 5C, top), but could reduce the level of secreted active MMP-9 protein (Revised Fig. 5C, bottom). This result indicated that MMP9 inhibitor SB-3CT may not affect the expression of MMP-9, but affect the activation of the protein.

**Reviewer #2:** Fig 5I: Hyperpermeability induced by NS1 alone is observed at only a single timepoint (7hrs), whereas the MMP9 treatment induces continual decrease in TEER starting at 3hrs. Adding MMP9 + NS1 had no effect greater than MMP9 alone. Altogether these data suggest that the hyperpermeability effect is entirely due to MMP9, without a contribution from NS1. (Line 272-273)

**Authors’ Response:** Thank you for the comment.

This is also the question we have addressed and discussed in the remaining part of the manuscript. NS1 could induce the permeability of endothelial cells. In our experiment, the addition of MMP-9 alone could also induce the permeability, but the expression of MMP-9 in endothelial cells is significantly lower than that in immune cells (Revised Fig. 4F). After being transmitted to the blood by mosquitoes, dengue virus first infected immune cells to produce MMP-9, and then reached the surface of endothelial cells to play a role under the interaction with NS1. Therefore, when NS1 and MMP-9 were added at the same time, its ability to induce permeability was still higher than MMP-9 alone (Revised Fig. 5J).

**Reviewer #2:** Fig 6A-C, Fig 8D-I: The effect DENV infection or NS1 on expression of cell junction proteins in mice does not obviously match the authors’ description (line 282-285, 334-341), e.g. ZO-2 in liver, b-catenin in spleen. This is not necessarily surprising, it would require a very strong effect to downregulate the total expression of these proteins in a full tissue.

**Authors’ Response:** Thank you for the comment.

To address this comment, we have repeated these experiments in the revised manuscript and used quantitative analyses of the levels of proteins expressed. The new results may provide more evidence to support our conclusions (Revised Fig. 6A–C and Revised Fig. 8D–F).

**Reviewer #3:** In the manuscript by Li and colleagues entitled, “DENV NS1 and MMP-9 cooperate to induce vascular leakage by altering endothelial cell adhesion and tight junction”, the authors describe potential mechanisms by which NS1 induces vascular leak that involve increased expression and activation of the matrix metalloprotease 9 (MMP-9) via NF-κB signaling pathways after DENV infection of human primary PBMCs or human monocytic cell lines (e.g. THP-1) in vitro. The authors also performed a series of experimental assays in vitro and in vivo to suggest that NS1 may interact with MMP-9 to facilitate disruption in the integrity of tight junctions, leading to endothelial hyperpermeability and vascular leakage in mouse tissues. This effect was inhibited in vitro by the specific inhibitor of MMP-9 SB-3CT and was relatively unaffected by NS1 in MMP9-/- mice, which suggests that MMP-9 is required for NS1-induced hyperpermeability.

**Authors’ Response:** Thank you very much for your comment.

**Reviewer #3:** The fact that the NS1 protein of flaviviruses can modulate the expression and activity of MMP-9 to perturb the integrity of biological barriers has been the focus of different groups, who have shown the effect of NS1 alone or NS1 in the context of viral infection (e.g., ZIKV) on the permeability of distinct biological systems such as the blood-testis barrier or the endothelial microvasculature (Chen et al., 2018; Hui et al., 2020).

**Authors’ Response:** Thanks for the comment

We have also noted that the paper you mentioned has published when our manuscript was in submission, which reported that MMP-9 plays an important role in endothelial cell permeability. We have cited this article in the discussion of the revised manuscript (Hui et al. Matrix metalloproteinase 9 facilitates Zika virus invasion of the testis by modulating the integrity of the blood-testis barrier. PLoS Pathog. 2020;16(4):e1008509.).

**Reviewer #3:** In the present study, authors present a significant set of biological and biochemical data explaining the potential NS1-induced mechanism leading to this MMP-9 phenomenon and its consequences on endothelial barrier function. Although the authors clearly demonstrate the requirement for MMP-9 in NS1-mediated endothelial dysfunction in vivo and in vitro (also shown by others), many of the mechanistic assertions from this study are not justified by the data. Therefore, certain additional experiments are required and questions and technical issues must be addressed, as outlined below in the sections that follow.

**Authors’ Response:** Thank you for the comments.

As you suggested, we have performed additional experiments to address technical issues in the revised manuscript (as outlined below in the sections that follow).

**Part II – Major Issues: Key Experiments Required for Acceptance**

**Reviewer #1**: 1) For argument one, it’s not clear how NS1 triggers NFkB activation. The experimental data was obtained from transfection experiments, it’s not clear whether NS1 is secreted in this system, and if not the secreted form (as suggested by the model), how would an ER-resident form of NS1 activate the NFkB pathway? NS1 has previously shown to activate other cytokines, is the mechanism here expected to be different?

**Authors’ Response:** Thanks for the comments.

According to your suggestion, we have carried out additional experiments to determine whether NS1 secrets in the cell systems in the revised manuscript. HEK293T cells, Hela cells, THP-1 differentiated macrophages, and HUVECs were transfected with plasmid pHA-NS1 at different concentrations. ELISA results indicated that the levels of NS1 protein in the supernatants of HEK293T cells, Hela cells, THP-1 differentiated macrophages, and HUVECs were increased as the concentrations of NS1 increased (Revised S1A–D Fig), demonstrating that NS1 protein is secreted in the supernatants of these cell systems.

It was reported previously that NS1 activates the NF-κB pathway (Hsu et al. Dengue virus infection induces interferon-lambda1 to facilitate cell migration. Sci Rep. 2016; 6: 24530. Silva et al., The dengue virus nonstructural protein 1 (NS1) increases NF-κB transcriptional activity in HepG2 cells. Arch Virol. 2011; 156(7): 1275-1279.). In our manuscript, we demonstrated that tagged NS1 activated NF-κB pathway through promoting phosphorylation of p65 and IKBα, and therefore, we speculated that DENV NS1 may activate cytokines through different mechanism.

**Reviewer #1**: 2) For argument two, how does NS1 decrease TIMP-1 production while simultaneously increase MMP-9 transcription? Isn’t TIMP-1 also a target gene for NFkB activation? Is the TIMP-1 reduction at the transcriptional, translation, or secretion level?

**Authors’ Response:** Thank you for the argument.

In the original manuscript, we have detected the level of secreted TIMP-1 protein in the supernatants of THP-1 cells and showed that TIMP-1 secretion was reduced by NS1 protein.

We have carried out additional experiments to address your comment in the revised manuscript. HEK293T cells, Hela cells, and THP-1 differentiated macrophages were transfected with plasmid pHA-NS1 at different concentrations. The new results showed that the levels of TIMP-1 protein were reduced by NS1 in HEK293T cells, Hela cells, and THP-1 differentiated macrophages in dose-dependent manners (S1E–G Fig), indicating that DENV NS1 inhibits TIMP-1 production in these cells. It was reported previously that TIMP-1 may be a target for NF-κB activation (Bai et al. Protective effects of calcitonin on IL-1 stimulated chondrocytes by regulating MMPs/TIMP-1 ratio via suppression of p50-NF-κB pathway. Biosci Biotechnol Biochem. 2019; 83(4):598-604.).

**Reviewer #1**: 3) For argument three, where do all these interactions supposed to occur to be relevant for the observed biological effect? The co-IP and co-staining are all intracellular, but the argument appears to be the secreted NS1 would be mediating effect on ECs in vivo as ECs are not readily infected. So the relevant interaction would be on the extracellular side. There is no data showing that extracellular NS1 having these interactions. Given that the form of the intracellular NS1 monomer/dimer is different from the extracellular hexamer, the binding data from the transfection experiments may not be relevant for pathogenesis in vivo. In Fig. 9, it’s not clear how the extracellular NS1-MMP-9 complex would have access to ZO-1.

**Authors’ Response:** Thanks for the comment.

According to your suggestion, we have performed additional experiments to confirm NS1/MMP-9 interaction in the revised manuscript.

Notably, His-pull-down assays demonstrated that purified DENV2 NS1 protein could directly interact with purified MMP-9 protein, but purified SARS-CoV-2 N protein failed to interact with purified MMP-9 protein (Revised Fig 2C), indicating that DENV2 NS1 protein specifically and directly interact with MMP-9 protein.

His-pull-down assays also demonstrated that purified DENV2-NS1 protein could directly interact with β-catenin and ZO-1, but purified SARS-CoV-2 N protein could not interact with β-catenin and ZO-1 in vitro (Revised Fig 7E, F).

**Reviewer #1**: 4) For argument three, if NS1 were to bridge an interaction between MMP-9 and the junction proteins, one would expect them to bind to different parts of NS1. This was not investigated. What about any mutant of NS1 that have been reported to lose the ability to cause EC hyperpermeability? Does the mutation affect any of the phenotypes reported here?

**Authors’ Response:** Thank you for the good comment.

In the original experiments, we have showed that NS1 interacts with the 106aa-440aa region of MMP-9.

According to your suggestion, we have constructed the truncated mutants of NS1 in the revised manuscript (Revised Fig. 7I). The new results from Co-IP analyses showed that all truncated NS1 proteins could interact with MMP-9 (Revised Fig. 7J). However, we noted that β-catenin could interact with the 212aa-352aa region of NS1 and ZO-1 could interact with the 282aa-352aa region of NS1 (Revised Fig. 7K). Additionally, we have checked relevant literatures and have not found any report on the role of specific structural domain of NS1 in EC permeability.

**Reviewer #1**: 5) In Fig. 6, the authors show that beta-catenin and the TJ proteins can be downregulated by MMP-9 alone, and to a lesser extent by NS1 alone. In fact, adding NS1 in this assay did not show more downregulation than MMP-9 alone. Then in Figure 7, the authors show that MMP-9 in unable to bind these junction proteins unless NS1 is present.

**Authors’ Response:** Thank you for the comment.

Sorry for the confusion. We might not describe the results clearly in the original manuscript. The difference between the two results may be due to the cell types we have used.

In Figure 6, HUVEC cells were used and MMP-9 is over-expressed in HUVEC cells. We have showed that MMP-9 alone could significantly down-regulate β-catenin and the TJ proteins (Revised Fig. 6G, lanes 3, 5, and 6). In Figure 7, HEK293T cells and Hela cells were used. We have demonstrated that NS1 recruits MMP-9 to interact with adhesion and tight junction proteins in HEK293T cells and Hela cells (Revised Fig. 7).

MMP-9 expression in endothelial cells is actually quite low compared with immune cells (Revised Fig. 4F). After being transmitted to the blood by mosquitoes, dengue virus first infected immune cells to produce MMP-9, and then reached the surface of endothelial cells to play a role in the interaction with NS1. We also showed that the level of permeability induced by NS1 and MMP-9 together was much higher than that by MMP-9 alone (Revised Fig. 6H and I).

**Reviewer #3**: 1. In Figure 1, the authors examined the levels of NS1, MMP-9, and TIMP-1 circulating in the sera of DENV-infected patients and healthy donors, finding that the levels of soluble NS1 were increased from day 2 until day 11, the last day of sample collection; however, the levels of MMP-9 seem to be significantly increased after 11 days. In patients experiencing severe dengue, increased vascular leak typically becomes clinically evident after 4-6 days of onset of symptoms, known as the critical phase. Can authors explain this apparent discordance in the timeline with respect to the appearance of these viral and host markers and the biological effect proposed by the study?

**Authors’ Response:** Thank you for your good question.

Yes, we have noted and showed that the levels of soluble NS1 were increased from day 2 until day 11, the last day of sample collection; however, the levels of MMP-9 seem to be significantly increased after 11 days. These results were pure testing data. We are sorry for that we cannot explain this apparent discordance in the timeline with respect to the appearance of these viral and host markers and the biological effect proposed by the study.

**Reviewer #3**: 2. In Figure 2, authors assessed the potential interaction between DENV protein(s), including NS1, and MMP-9 in co-transfected 293T cells by performing a co-immunoprecipitation (Co-IP) assay using anti-Flag antibody. Authors should perform Co-IP experiments using either an anti-NS1 or anti-MMP9 mAb. Is this pulled-down MMP-9 more active after interaction of NS1?

**Authors’ Response:** Thank you for your comment.

According to your suggestion, we have performed additional experiments to confirm NS1/MMP-9 interaction in the revised manuscript.

Notably, His-pull-down assays demonstrated that purified DENV2 NS1 protein could directly interact with purified MMP-9 protein, but purified SARS-CoV-2 N protein failed to interact with purified MMP-9 protein (Revised Fig 2C), indicating that DENV2 NS1 protein specifically and directly interact with MMP-9 protein.

His-pull-down assays also demonstrated that purified DENV2-NS1 protein could directly interact with β-catenin and ZO-1, but purified SARS-CoV-2 N protein could not interact with β-catenin and ZO-1 in vitro (Revised Fig 7E, F).

**Reviewer #3**: 3. The authors claim that an interaction between NS1 and MMP9 results in NS1-mediated TJ/AJ disruption, and while the data are certainly suggestive, they are certainly not conclusive. To make this assertion, the authors must demonstrate that disrupting this interaction ablates NS1-mediated pathology; this can be done using mutants of either NS1 or MMP9.

**Authors’ Response:** Thank you for your comment.

We have proved the interaction between NS1 and 106a-440aa of MMP-9 (Revised Fig. 2E). According to your suggestion, we have performed additional experiments to demonstrate that disrupting interaction ablates NS1-mediated TJ/AJ disruption in the revised manuscript. HUVEC cells were co-transfected with NS1 plus MMP9-D2, NS1 plus MMP9-D3, or NS1 plus MMP-9. The results showed that the TJ/AJ disruption was significantly induced by NS1 plus MMP-9, but the TJ/AJ disruption was relatively unaffected by NS1 plus MMP9-D2 or NS1 plus MMP9-D3 (Revised S4B Fig), indicating that the interaction of MMP9 and NS1 plays an important role in S1-medited TJ/AJ disruption.

**Reviewer #3**: 4. Further, the authors claim that TIMP-1 may be playing a role in regulating MMP-9 activity. How do the authors propose this to be the case? Either way, the authors do not functionally demonstrate a relationship between TIMP1/NS1 and MMP9. If the authors want to assert this point, they must provide further experiments.

**Authors’ Response:** Thank you.

In our original manuscript, the relationship between the TIMP1/NS1 and MMP-9 was demonstrated by analyzing the patients’ serum levels.

According to your suggestion, we have carried out additional experiments to demonstrate the relationship between the TIMP1/NS1 and MMP-9. HEK293T cells, Hela cells, and THP-1 differentiated macrophages were transfected with plasmid pHA-NS1 at different concentrations. The new results showed that the levels of TIMP-1 protein were reduced by NS1 in HEK293T cells, Hela cells, and THP-1 differentiated macrophages in dose-dependent manners (S1E–G Fig), indicating that DENV NS1 inhibits TIMP-1 production in these cells.

**Reviewer #3**: 5. The IFA in Figure 2C displays blown-out cytoplasmic signal and thus no conclusion of colocalization can be made. Please present an image with less blown-out signal.

**Authors’ Response:** Thank you for the suggestion.

According to your suggestion, we have presented a better image with less blown-out in the revised manuscript (Revised Fig 2D).

**Reviewer #3**: 6. It is well appreciated that Co-IP assays have a propensity to yield false positives, especially when overexpressing proteins in 293T cells. The authors should demonstrate interaction of endogenous proteins in infected THP1 or other cells (ideally, the authors can demonstrate an interaction by pulling down either protein). Further, it would be best to demonstrate the interaction in an orthogonal assay. On the same point, IFA colocalization studies between all proteins reported to interact would be helpful in interpreting the data.

**Authors’ Response:** Thank you for the comment.

According to your suggestion, we have performed additional experiments to confirm NS1/MMP-9 interaction in the revised manuscript.

Notably, His-pull-down assays demonstrated that purified DENV2 NS1 protein could directly interact with purified MMP-9 protein, but purified SARS-CoV-2 N protein failed to interact with purified MMP-9 protein (Revised Fig 2C), indicating that DENV2 NS1 protein specifically and directly interact with MMP-9 protein.

His-pull-down assays also demonstrated that purified DENV2-NS1 protein could directly interact with β-catenin and ZO-1, but purified SARS-CoV-2 N protein could not interact with β-catenin and ZO-1 in vitro (Revised Fig 7E, F).

**Reviewer #3**: 7. All Western blot data showed in Figure 2 correspond to anti-HA and anti-Flag antibodies. As Pro- vs Active MMP-9 have different molecular weights, what is the molecular weight of the MMP-9 shown in Figure 2? And in the other Figures throughout the manuscript?

**Authors’ Response:** Thank you.

The molecular weight of MMP-9 is 92KDa.

**Reviewer #3**: 8. In Figure 4, the authors describe a series of data regarding how DENV2 induces MMP-9 expression and secretion in human PBMCs and macrophages but not in HUVECs. DENV infection of human endothelial cells has been always a controversial topic, as these cells are not considered a target for DENV replication in vivo as monocytes and other immune cells are. Interestingly, here the data showed that indeed HUVECs are not significantly infected with DENV during the timeline of the experiment (24 hours) and thus, changes in the expression of MMP-9 were not detected. Along these lines, can the authors overexpress NS1 in HUVECs (or treat with secreted NS1) to assess whether this alters MMP-9 expression?

**Authors’ Response:** Thank you for the comment.

According to your suggestion, we have performed additional experiments to assess the role of NS1 in MMP-9 expression in HUVECs.

HUVECs were transfected with plasmid pHA-NS1 at different concentrations. ELISA results indicated that the levels of NS1 protein in the supernatants of HUVECs were increased as the concentrations of NS1 increased (Revised S1D Fig), demonstrating that NS1 protein is secreted in the supernatants of these cell systems. We also showed that level of MMP-9 protein was relatively unaffected by NS1 protein in HUVECs (Revised S1H Fig).

**Reviewer #3**: 9. What were the levels of NS1 expression on THP-1 cells infected with DENV? This piece of data is missing, and it is important to show that levels of NS1 may correlate with the levels of MMP-9 expression in infected cells as shown in Figure 3A, where THP-1 cells were transfected with NS1.

**Authors’ Response:** Thank you for the comment.

In the original manuscript, we have not checked the levels of NS1 expression on THP-1 cells infected with DENV.

According to your suggestion, we have performed additional experiments to determine the levels of NS1 expression on THP-1 cells infected with DENV in the revised manuscript.

PMA-differentiated THP-1 macrophages were infected with DENV2 for different times, as indicated. The new results showed that the secretion of NS1 in supernatants (Revised Fig 5A, top) the production of MMP-9 protein and NS1 protein in cell lysates (Revised Fig 5A, bottom) were up-regulated in time-dependent fashions.

**Reviewer #3**: 10. In Figure 5, the authors showed that supernatants collected from DENV-infected THP-1 cells increased the permeability of naïve HUVEC monolayers, which was prevented by an MMP-9-specific inhibitor (SB-3CT). There are several points that need to be clarified here: 1) Did authors measure the levels of NS1 present in the supernatant collected from DENV-infected THP-1 cells that were added on top of HUVEC monolayers? This is important to specify, as soluble NS1 can also modulate the permeability of HUVEC monolayers. 2) The authors must use an irrelevant chemical inhibitor to demonstrate the specificity of the TEER inhibition showed in Figure 5A once SB-3CT was mixed with the infected supernatant.

**Authors’ Response:** Thanks.

According to your suggestion, we have carried out additional experiments to clarify the levels of NS1 presented in the supernatant collected from DENV-infected THP-1 cells in the revised manuscript. PMA-differentiated THP-1 macrophages were infected with DENV2 for different times, as indicated. The new results showed that the secretion of NS1 in supernatants (Revised Fig 5A, top) and the production of MMP-9 protein and NS1 protein in cell lysates (Revised Fig 5A, bottom) were up-regulated in time-dependent fashions.

Additionally, according to your suggestion, we have performed additional experiments by adding NF-KB inhibitor SC75741 as an irrelevant chemical inhibitor to DENV-infected THP-1 supernatant. HUVECs grown on polycarbonate membrane system were incubated with the supernatants of DENV2-infected HUVEC cells or DENV2-infected THP-1 differentiated macrophages or pre-incubated with SB-3CT (a specific inhibitor of MMP-9) or irrelevant chemical inhibitor SC75741 (a specific inhibitor of NF-κB). Endothelial permeability was evaluated by measuring trans-endothelial electrical resistance (TEER) (ohm) using EVOM2 epithelial volt ohm meter. The level of TEER was not affected by the supernatants of DENV-infected HUVECs; significantly attenuated by the supernatants of DENV-infected THP-1 differentiated macrophages from 3 h to 15 h post-treatment; and however, such reduction was recovered by the treatment of SB-3CT, but not recovered by the treatment of SC75741 (Revised Fig 5B); suggesting that MMP-9 plays an important role in the induction of endothelial hyperpermeability mediated by DENV infection.

**Reviewer #3**: 11. In Figure 5B, the authors examined the effect of the MMP-9-specific inhibitor SB-3CT in IFNAR-/- mice infected with DENV2 and then measured, among other things, the amount of MMP-9 circulating in the sera of the experimental mice. Do authors know if these types of inhibitors may have pleiotropic effects in vivo that may have affected virus replication? Also, what were the viremia levels in DENV-infected mice treated and not treated with this inhibitor? This is not shown in Figure 5.

**Authors’ Response:** Thank you for the comment.

We might not describe it clearly in the original manuscript. We have detected the level of viremia in the blood of DENV-infected mice. IFNAR^-/-^ C57BL/6 mice were treated with PBS as a control group (n = 4), infected with DENV2 (n = 6), and intravenously treated with MMP-9 specific inhibitor SB-3CT and then infected with DENV2(NGC) (n = 6). DENV2 E and NS5 RNA were detected at high levels in the blood of DENV2-infected mice or SB-3CT-treated and DENV2-infected mice at 2 days and 4 days post-infection, but not detected in the blood of mocked-infected mice indicating that DENV2 replicated well in the mice (Revised S3A and B Fig). We also noted that compared with DENV2-infected mice, the DENV2 E and NS5 RNA has no influence in SB-3CT-treated and DENV2-infected mice, indicating that SB-3CT had no effect on the replication of virus in mice (Revised S3A and B Fig). The results showed that DENV+SB-3CT group had no effect on the expression of E and NS5 genes of DENV compared with the DENV-infected group, indicating that SB-3CT maybe had no effect on the viremia of mice.

**Reviewer #3**: 12. As suggested above for Figure 5A, authors must include irrelevant (unrelated) chemical inhibitor controls in these TEER experiments (Figure 5I).

**Authors’ Response:** Thank you.

According to your suggestion, we have performed additional experiments by adding NF-KB inhibitor SC75741 as an irrelevant chemical inhibitor. HUVECs grown on Transwell inserts were incubated with purified MMP-9 protein, NS1 protein, and NS1 protein plus MMP-9 protein at different concentrations, or pre-treated with SB-3CT or irrelevant chemical inhibitor SC75741 and then incubated with NS1 protein plus MMP-9 protein. The level of TEER was reduced by MMP-9 protein alone, NS1 protein alone, and MMP-9 protein plus NS1 protein from 2 to 7 h post-treatment; but the reductions were recovered by SB-3CT and not restored by SC75741 (Revised Fig 5J); demonstrating that MMP-9 induces endothelial hyperpermeability in human endothelial cells. The new results showed that NF-KB inhibitor SC75741 could not restore the effect on TEER value compared with SB-3CT.

**Reviewer #3**: 13. In Figure 6A-C and G, the Western blot data is exceedingly difficult to interpret as bands corresponding to tight or adherens junction proteins look shortened and sometimes fussy. Authors should repeat these Western blots to show better images and include a panel with quantitative analysis of the protein expression.

**Authors’ Response:** Thank you for the comment.

According to your suggestion, we have repeated the experiments, showed better images, and included a panel with quantitative analysis of the protein expression in the revised manuscript.

The results showed that E-cadherin, β-catenin, and ZO-2 proteins were down-regulated by DENV infection compared with mock-infected mice in the liver (Revised Fig 6A), spleen (Revised Fig 6B), and lung (Revised Fig 6C), but rescued in SB-3CT-treated and DENV-infected mice (Fig 6A–C).

The adhesion junction proteins such as N-cadherin and β-catenin, and the tight junction factors including Claudin-1 and Zona Occludens proteins (ZO-1, ZO-2, and ZO-3, also known as tight junction proteins, TJP-1, TJP-2, and TJP-3) were little affected by only NS1 protein in Hela cells (Revised S4 Fig) or HUVECs (Revised Fig 6G). In contrast, the tight junction protein ZO-1, ZO-2 and the adhesion junction protein β-catenin, N-cadherin were reduced by MMP-9 (2 μg) or NS1 (1 μg) plus MMP-9 (1 μg), and such reductions were eliminated by SB-3CT in both Hela (Revised S4 Fig) and HUVEC cells (Revised Fig 6G).

**Reviewer #3**: 14. Figure 6D-F, in which authors aim to show the expression/staining of beta-catenin, are not interpretable. Authors should perform some type of protein quantification.
**Authors’ Response:** Thank you.

As you suggested, we have performed quantitative analyses of the images in the revised manuscript (Revised Fig. 6D–F).

**Reviewer #3**: 15. In Figure 8A-C, authors show very nicely the effect of NS1, MMP-9 and the knockout MMP-9-/- on vascular leak detected in mice. However, again the Western blot data in panels E and F are not easy to interpret, particularly for ZO-2 and beta-catenin proteins. The authors should repeat this and show quantitative analysis of the protein expression.

**Authors’ Response:** Thank you for the comment.

According to your suggestion, we have repeated the experiments and carried out quantitative analyses of the protein expression in the revised manuscript (Revised Fig. 8D–F). β-catenin and ZO-2 proteins were down-regulated by NS1 in the lung (Revised Fig 8D), spleen (Revised Fig 8E), and liver (Revised Fig 8F) of C57BL/6 mice, relatively unaffected by NS1 in MMP9^-/-^ mouse tissues, and significantly reduced by NS1 in the tissues of MMP9^-/-^ mice treated with MMP-9 protein (Revised Fig 8D–F).

**Part III – Minor Issues: Editorial and Data Presentation Modifications**

**Reviewer #1**: 1) This group previously published a similar study on DENV M Protein, which they show that it can induce vascular leakage in mice by interacting with the NLRP3 Inflammasome. Animal model data was also presented in that paper. That study doesn’t appear to be cited or discussed here. Which mechanism do the authors believe is more important?

**Authors’ Response:** Good suggestion.

We have citated the previously published article and discussed it in the revised manuscript.

We speculate there may be two different molecular mechanisms: one is that M protein activates the NLRP3 inflammasome, causing high levels of inflammatory response and resulting in vascular leakage; the another is that NS1 protein recruits MMP-9 protein through degrading TJ/AJ protein to directly damage the permeability of endothelial cells.

**Reviewer #1**: 2) There is an issue of missing controls and data quantification. Are the NFkB activation or MMP-9 bindings specific to DENV NS1 or other flavivirus NS1 can also do this? I think it would be important to investigate this if conclusions were to be drawn for dengue pathogenesis. For NF-kB activation, what about other targets or non-target genes?

**Authors’ Response**: Thanks.

A recently published paper demonstrated that ZIKV NS1 protein plays an important role in ZIKV infection crossing the blood-testis barrier through K63 ubiquitination of MMP-9 (Hui et al. Matrix metalloproteinase 9 facilitates Zika virus invasion of the testis by modulating the integrity of the blood-testis barrier. PLoS Pathog. 2020;16(4): e1008509.). For NF-κB activation, it had previously reported that DENV NS5 could induce the expression of RANTES through the NF-κB pathway (Khunchai et al. NF-κB is required for dengue virus NS5-induced RANTES expression. Virus Res. 2015;197:92-100.) and DENV NS1 protein activates NF-κB transcription (Silva et al. The dengue virus nonstructural protein 1 (NS1) increases NF-κB transcriptional activity in HepG2 cells. Arch Virol. 2011;156(7):1275-1279.). However, whether other targets or non-target genes involved in NF-κB activation remains to be further determined.

**Reviewer #1**: 3) Fig. 5I. TEER values should be shown. These values for HUVEC are typically very low to begin with, a 10-20% reduction may not be that relevant for barrier function.

**Authors’ Response**: Thank you for the comment.

As we known, it has been reported that the reduction of TEER value by 10-20% can represent endothelial cell for barrier function (Puerta-Guardo et al. Dengue Virus NS1 Disrupts the Endothelial Glycocalyx, Leading to Hyperpermeability. PLoS Pathog. 2016;12(7):e1005738. Glasner et al. Dengue virus NS1 cytokine-independent vascular leak is dependent on endothelial glycocalyx components. PLoS Pathog. 2017;13(11):e1006673.).

**Reviewer #1**: 4) Fig. 8D. ZO-2 is similarly downregulated by NS1 regardless of MMP-9 was knocked out or not.

**Authors’ Response:** Thank you for the comment.

According to your suggestion, we have repeated the experiment and carried out quantitative analysis of the protein expression in the revised manuscript. The new results clearly showed that ZO-2 was downregulated by NS1 in WT mice lung, but relatively unaffected by NS1 in MMP-9 knocked out mice lung (Revised Fig. 8D).

**Reviewer #1**: 5) The paragraph title: “DENV enhances the production of MMP9 and TIMP-1 in severe dengue patients” is incorrect. TIMP-1 production decreases in patients as well as in vitro with NS1 expression.

**Authors’ Response:** Thank you very much for the comment.

As you suggested, we have changed the incorrect paragraph title “DENV enhances the production of MMP9 and TIMP-1 in severe dengue patients” to a correct one “DENV enhances MMP9 production and attenuates TIMP-1 expression in severe dengue patients” in the revised manuscript.

**Reviewer #1**: 6) Line 157, “converse correlation” is incorrect, should be “inverse correlation”

**Authors’ Response**: Thank you.

We have changed “a converse correlation” to “an inverse correlation” in the revised manuscript (Revised manuscript, line 162).

**Reviewer #1**: 7) Line 308, “failed to interaction with” should be “failed to interact with”.

**Authors’ Response:** Good questions. According to your suggestion, we used “failed to interact with” instead of “failed to interaction with” in revised manuscript (Revised manuscript, line 343).

**Reviewer #2**: Line 51-52: the claim that the proposed mechanism is “the main reason leading to death in severe dengue patients” should be toned down.

**Authors’ Response:** Thank you for the suggestion.

According to your suggestion, we have changed the whole sentence “We found that a distinct mechanism of DENV NS1 and MMP-9 cooperatively induce vascular leakage is the main reason leading to death in severe dengue patients.” to “We revealed a possible mechanism by which DENV NS1 and MMP-9 cooperatively induce vascular leakage, which may be one of the reasons leading to death in severe dengue patients.” in the revised manuscript (Revised manuscript, lines 52-53).

**Reviewer #2**: Line 105-106: As written this implies that facilitating tumor migration is the normal physiologic function of MMP9.

**Authors’ Response:** Thanks.

As you suggested, we have changed the description from “the MMP-9 protein specifically degrades extracellular matrix to promote tumor migration” to “the MMP-9 protein could promote tumor migration by degrading extracellular matrix” in the revised manuscript (Revised manuscript, lines 108-109).

**Reviewer #2**: Fig 1: For D,E,F, re-orient graphs so independent variable (D NS1, E TIMP1, F NS1) is on the X axis and dependent variable on the y axis.

**Authors’ Response:** Thank you for your suggestion.

According to your suggestion, we have changed the Figure 1D–F to conform to the re-orient graphs so independent variable (D NS1, E TIMP1, F NS1) is on the X axis and dependent variable on the y axis.

**Reviewer #2**: The manuscript would benefit from another round of editing to polish the writing.

**Authors’ Response**: Thank you.

We have made some editing to polish the writing carefully in the revised manuscript.

**Reviewer #3**: 1. There are many grammatical errors and typos throughout the manuscript. Please revise carefully.

**Authors’ Response:** Thank you.

We have corrected some errors and typos in the revised manuscript.

**Reviewer #3**: 2. Could the authors specify which serotype of DENV NS1 is purchased from the Native Antigen Company?

**Authors’ Response**: Thanks.

The serotype of DENV NS1 purchased from the Native Antigen Company is DENV-2 serotype.

**Reviewer #3**: 3. The MMP9/NS1 connectionis not a new finding, please cite relevant literature to describe what is reported previously (2-3 papers at least).

**Authors’ Response:** Thank you for the suggestion.

As you suggested, we have cited 2 relevant references in the revised manuscript (Chen et al. Macrophage migration inhibitory factor is critical for dengue NS1-induced endothelial glycocalyx degradation and hyperpermeability. PLoS Pathog. 2018;14(4):e1007033. Hui et al. Matrix metalloproteinase 9 facilitates Zika virus invasion of the testis by modulating the integrity of the blood-testis barrier. PLoS Pathog. 2020;16(4):e1008509.).

**Reviewer #3**: 4. Author summary line 51-52: The authors write, “We found that a distinct mechanism of DENV NS1 and MMP-9 cooperatively induce vascular leakage is the main reason leading to death in severe dengue patients.” This statement is a wild overstatement that is not supported by the data. It is also likely not accurate, as vascular leakage in severe dengue disease is undoubtedly multifactorial and complex.

**Authors’ Response:** Thank you for the comment.

According to your suggestion, we have changed the whole sentence “We found that a distinct mechanism of DENV NS1 and MMP-9 cooperatively induce vascular leakage is the main reason leading to death in severe dengue patients.” to “We revealed a possible mechanism by which DENV NS1 and MMP-9 cooperatively induce vascular leakage, which may be one of the reasons leading to death in severe dengue patients.” in the revised manuscript (Revised manuscript, lines 52-53).

**Reviewer #3**: 5. Figure 4E, label on y-axis is missing.

**Authors’ Response**: Thanks.

According to your suggestion, we have added the Y-axis label of Figure 4E in the revised manuscript (Revised Fig 4E).

**Reviewer #3**: 6. Figure 5B, label on y-axis is missing.

**Authors’ Response:** Thanks again.

According to your suggestion, we have added the Y-axis label of Figure 5B in the revised manuscript (Revised Fig 5C).

**Reviewer #3**: 7. Figure 5F, “live” instead of “liver”.

**Authors’ Response**: Thank you.

According to your suggestion, we have changed “live” to “liver” in the revised manuscript (Revised Figure 5G).
