# Supplementary material for: DENV NS1 and MMP-9 cooperate to induce vascular leakage by altering endothelial cell adhesion and tight junction
Source: PLoS Pathog. 2021 Jul 26;17(7):e1008603. doi: 10.1371/journal.ppat.1008603 (PMC8341711; doi:10.1371/journal.ppat.1008603)
Supplement: Attachment — Submitted filename: PLOS Pathog Responses to the Reviews Pan-Wu 20210125.docx [file ppat.1008603.s014.docx]

**Responses to the Reviews’ Comments**

**Part I – Summary**

Please use this section to discuss strengths/weaknesses of study, novelty/significance, general execution and scholarship.

**Reviewer #2:** In this revised manuscript, Pan and colleagues have made revisions and added new data to respond to reviewer comments. Many reviewer concerns have been addressed.

**Authors’ Response:** Thank you very much for your summary on the revised manuscript.

**Reviewer #3**: This revised manuscript by Pan et al., addresses some of my original comments, but a number of critical questions remain. As the functional relevance of MMP9 in NS1-mediated vascular leak has been previously reported, the novel elements of this manuscript are the mechanistic interaction data. As such, the most critical points the authors still need to address include demonstrating the functional relevance of the interactions between NS1 and MMP9 and demonstrating the protein-protein interactions (between NS1 and MMP9) using an orthogonal approach to the current co-immunoprecipitation assays, which are not fully convincing on their own. Further, although the correlations are interesting, the authors’ assertion that TIMP1 plays a role in their model must be functionally validated or else should be de-emphasized. The authors should address the points below.

**Authors’ Response:** Thank you very much for your comments.

As you suggested, we have performed additional relevant experiments in the revised manuscript to further demonstrate the interaction between NS1 and MMP9 by using orthogonal methods, including BiFC assay (Revised Fig. 2C) and yeast double hybridization assay (Revised Fig. 2E). In addition, according to your comment, we have deleted the results regarding TIMP1 in the revised manuscript. Moreover, as you requested, we have addressed the points that you have raised and have carefully modified the manuscript in the revisions.

**Reviewer #4**: The manuscript attempts to define how NS1 induces EC permeability in dengue infections. The authors have identified MMP-9 as an important modulatory of permeability by NS1. This is important. Information but has been shown previously. The mechanistic data as to how NS1 does this uses several different cells lines which makes the manuscript confusing and fails to account for changes in structure of the form of NS1 both inside and outside the cell. There is some concern that the HUVEC data are not robust as the authors appear to have not created tight barriers to address the questions.

**Authors’ Response:** Thank you very much for the comments.

Our studies demonstrated that DENV NS1 regulates MMP9 to induce vascular leakage. To further prove the hypothesis, we used different cell lines, including HEK293T, Hela, THP-1, and HUVEC. Among them, HEK293T cells and Hela cells are easily transfected with plasmids, and the main purpose of whose are to verify the protein expression and the interaction between proteins. THP-1 cells and HUVEC cells were mainly used to study DENV virus infection and endothelial cell permeability. By using different cell lines, we should make the experimental results more sufficient and reliable. Previous studies have shown that NS1 exists in the form of dimer in the cell and hexamers outside the cell. Our experiments proved that NS1 could be expressed in the cell and secreted outside the cell (Revised Fig. S1). HUVECs have been used previously to detect changes in endothelial cell permeability (Beatty et al., Dengue virus NS1 triggers endothelial permeability and vascular leak that is prevented by NS1 vaccination. Sci Transl Med 2015; 7(304): 304ra141; Pan et al., Dengue virus infection activates interleukin-1β to induce tissue injury and vascular leakage. Front Microbiol 2019; 10: 2637.). According to your suggestions, we repeated the relevant experiments and described the using method of HUVEC cells in detail in the revised manuscript.

**Part II – Major Issues: Key Experiments Required for Acceptance**

Please use this section to detail the key new experiments or modifications of existing experiments that should be absolutely required to validate study conclusions.

Generally, there should be no more than 3 such required experiments or major modifications for a "Major Revision" recommendation. If more than 3 experiments are necessary to validate the study conclusions, then you are encouraged to recommend "Reject".

**Reviewer #3**: 1. MMPs are secreted as inactive proenzymes and are activated by the cleavage of the propeptide domain by proteolytic enzymes. In this study, one of the main conclusions suggested by the authors is that NS1 interacts with the MMP9 expressed in immune cells, leading to degradation of tight and adherens junction proteins on endothelial cells. However, what is the result of this interaction? Does NS1 modulate the proteolytic activity of MMP9, leading to its activation? Authors must experimentally demonstrate this phenomenon.

**Authors’ Response:** Thank you very much for the comments and questions.

In order to address your questions, we have performed additional experiments to determine the role of interaction of NS1 with MMP9 in the regulation of MMP9 gelatinase activity in the revised manuscript. The new results from gelatinase activity assays showed that gelatinase activity was only detected in the presence of both NS1 protein and pro-MMP9 protein, but the enzyme activity was not detected in the presence of BSA protein, NS1 protein, or pro-MMP9 protein alone, or in the presence of BSA and pro-MMP9 (Revised Fig. 3H). This result demonstrated that NS1 regulates the protease activity of MMP9 by interacting with MMP9.

2. Most importantly, regarding the new data in fig. S4B, MMP9 truncation mutants cannot be used to make a functional conclusion regarding whether the interaction between MMP9 and NS1 is critical for NS1-mediated cell-cell junction disruption, as the loss of this phenotype can be explained either by the loss of interaction between NS1 and MMP9 or simply by the loss of protein function due to MMP9 truncation. The authors would need to create point mutants of MMP9 and/or NS1 that no longer interact and demonstrate that these mutants do not mediate intercellular junction disruption.

**Authors’ Response:** Very good suggestion and comment.

MMP9 mutants D2 and D3 may indeed lost the function of MMP9 protein (Fig. S4B). We tried to construct a series of truncated mutation of NS1, however, all of the truncated mutations interacted with MMP9 (Original Fig. 7J, now Revised S6C Fig). According to your suggestion, we have constructed a series of truncated mutants of NS1 according to the domain of NS1 in the revised manuscript. Similarly, the new Co-IP results also showed that different domains of NS1 could interact with MMP9 (Revised S6E Fig). Although your suggestion is very good, we could not to create point mutants of MMP9 and/or NS1 that no longer interact and demonstrate that these mutants do not mediate intercellular junction disruption.

3. The data in Figure 7J are confusing, as it appears that all truncation mutants of NS1 interact with MMP9, suggesting that this interaction may not be specific, which is not uncommon in CO-IP assays. Can the authors explain these data?

**Authors’ Response:** Good question and comment.

According to your suggestion, we have constructed the truncated NS1 protein by gradually reducing 70 amino acids in order to define the interaction region between NS1 and MMP9. For our surprise, after repeated this experiment, we found that all the truncation of NS1 interacted with MMP9. During this experiment, we used the empty plasmid that did not express NS1 protein as the negative control, and found that it could not precipitate with MMP9 protein (Original Fig. 7J, now Revised S6C Fig), indicating that our experimental system was no problem. To further prove this conclusion, we have constructed the truncated mutants of NS1 according to three different domains of NS1. Similar to the previous results, the new results showed that domain 2 and domain 3 of NS1 could interact with MMP9. However, domain 1 of NS1 failed to be precipitated with MMP9, which may due to it is too small. In this experiment, we also used the empty plasmid that did not express NS1 protein as the negative control, and found that it could not precipitate with MMP9 protein (Revised S6E Fig). Taken together, above results demonstrated that our experimental system was not problematic.

4. To confirm the specificity of the interaction of NS1 with MMP9, the authors should utilize an orthogonal method (e.g., Biomolecular fluorescence complementation (BIFC), yeast 2-hybrid, or through convincing subcellular colocalization).

**Authors’ Response:** Thank you for the very good suggestion.

According to your suggestion, we have performed additional experiments to confirm the specific interaction between NS1 and MMP9 by using orthogonal methods, including BiFC assay (Revised Fig. 2C) and yeast double hybridization assay (Revised Fig. 2E) in the revised manuscript. The new results confirmed that NS1 interacted with MMP9 (Revised Fig. 2C and 2E).

5. If the proposed model is correct, the authors should be able to visualize NS1 and MMP9 colocalizing at cell-cell junctions when added to cells exogenously. Such an observation is critical to support the model proposed by the authors. To clarify, the overexpression data in Fig. 2D demonstrate broad cytoplasmic localization of overexpressed proteins and not colocalization (as the signal of both proteins is everywhere in the cytoplasm).

**Authors’ Response:** Very good suggestion.

According to your suggestion, we have supplemented new experiments in the revised manuscript. The new results from immunofluorescence analysis showed that in HEK293T cells (Fig. 7H) and Hela cells (Fig. 7I) co-transfected with HA-MMP-9, GFP/HA-MMP-9, GFP-NS1/HA-MMP-9, Flag-β-catenin, Flag-β-catenin/GFP-NS1/HA-MMP-9, Flag-ZO-1 or Flag-ZO-1/GFP-NS1/ HA-MMP-9, NS1 and MMP9 could colocalize at cell-cell junctions (β-catenin/ZO-1). Since we have the new results, the previous results of Fig. 2D have been removed from the revised manuscript.

6. As no function is demonstrated for TIMP1, the authors need to experimentally demonstrate why this is important or remove from their central mechanistic message (refer to the association result).

**Authors’ Response:** Thank you for the comment.

We agree that although we showed that TIMP-1 is involved, the function of TIMP1 is not demonstrated. Therefore, we have deleted the preliminary results of TIMP1 in the revised manuscript.

**Reviewer #4:** In figure 1. The authors state they are severe dengue patents. How is severity classified? Were they all DHF or DSS patients? The authors should include both primary and secondary dengue patients and both severe and non-severe to truly understand how NS1 is modulating the level of TIMP-1 and MMP-9. If MMP-9 and TIMP-1 are important for severity they should not be changed in non-severe dengue.

**Authors’ Response:** Thank you for the comments.

As you suggested, we have added the description of the patient information in the revised manuscript (Revised S1 Table). In this study, severe dengue patient samples were collected at the Eight People’s Hospital of Guangzhou during a DENV outbreak in Guangzhou, China, in 2014. The typical clinical characteristics of these severe patients are positive for Dengue virus nucleic acid test, persistent fever, rapid reduction of platelets, and fatigue, muscle aches, skin rashes, and bleeding. They are all DHF patients but not DSS patients. According to the DENV antibody of IgG and IgM, only one (DV-8) of these severe patients was secondary Dengue patients. It has been proved that MMP-9 is also increased in non-severe Dengue patients (Chen et al., Macrophage migration inhibitory factor is critical for dengue NS1-induced endothelial glycocalyx degradation and hyperpermeability. PLOS Pathog 2018; 14: e1007033).

Furthermore, given that the clinical samples had both IgG and IgM levels, classification of primary or secondary is possible based on this data. As is the determination if NS1 increases over time due to an increase in virus levels (as we know it does), as the methods state that qPCR for DENV viral load was performed and the data are then available. Contrary to the response to Reviewer 2 when asked for this data. These data need to be included.

**Authors’ Response:** Good suggestion.

According to your suggestion, in the revised manuscript, we have analyzed the IgG and IgM levels of the patients and showed that only one of the 8 patients was secondary Dengue patients based on the IgG levels (Revised S1 Table). We are sorry that the description of qRT-PCR to quantify the DENV viral load in the original Methods section was inappropriate. The hospital only qualitatively provided those patients with positive nucleic acid test for DENV, but did not give a specific quantitative value. The hospital only kept serum samples from the patients when the samples were collected in 2014. We have re-measured the level of NS1 in the sera (Revised Fig. 1A). According to your suggestion, we have tried to extract RNA from the serum samples, but unfortunately, we did not detect DENV viral copies through qRT-RCR. Thus, we are to say that we could not prove the data of DENV viral load.

It would also be useful to have each individual patient marked with a different symbol so that they can be followed specifically in A B and C. Do all patients show this increase? Or only some? Is there a correlation to viral load?

**Authors’ Response:** Good suggestion.

According to your suggestion, we have added the information of the expression levels of NS1 and MMP9 of each patient (Revised S2 Table). The results showed that 6 of 8 patients marked with a different symbol (Revised Fig. 1A and 1B). Again, because we do not have the information of DENV viral load, the correlation NS1 and MMP9 levels to viral load could not be obtained.

In figure 3. Given that Dengue does not infect endothelial cells in vivo, expressing NS1 in HUVECs to determine if it is secreted is not relevant.

**Authors’ Response:** Good comment.

Sorry for the confusion. As we demonstrated that NS1 could induce the expression of MMP9 by activating the NF-κB signaling pathway (Revised Fig. 3), thus we further determined the ability of DENV to infect HUVEC and THP-1 cells. Our results showed that DENV could infect THP-1 cells (Revised Fig. 4A, top and 4F) and HUVEC cells (Revised Fig. 4A, bottom and 4F). However, unlike in DENV-infected THP-1 cells (Revised Fig. 4B, C, and G), DENV-infected HUVEC cells displayed no effect on MMP9 expression (Revised Fig. 4D, E, and G). We wanted to further prove the expression of transfected NS1 plasmid in HUVEC cells and assess that overexpress NS1 in HUVECs whether alters MMP-9 expression. Thus, we have supplemented the overexpression of NS1 in HUVECs here to answer this question.

There is no information in the methods detailing how HUVECs were transfected, or indeed any of the cells used in the study. HUVECs are incredibly difficult to transfect and keep alive and this information needs to be included.

**Authors’ Response:** Thank you for the good comment.

As you suggested, we have supplemented the information regarding HUVECs transfection in the Methods section of the revised manuscript.

It is true that HUVECs are difficulty to be transfected. The transfection procedure was as follows: when HUVEC cells grew to 80-90% of the volume of the cell culture dish, the medium were replaced with serum-free medium, and transfected with the ratio of plasmid concentration (1 μg) and Lipo2000 dosage 1:3. After transfection, HUVEC cells were replaced with complete medium until the predetermined time point. In our manuscript, we demonstrate the molecular mechanism by which DENV NS1 regulates MMP9 to induce vascular leakage through a series of experiments. To prove our hypothesis, we tested it in different cell lines, including HEK293T, Hela, THP-1, and HUVEC. Among them, HEK293T and Hela cells are easy to be transfected with plasmids, and the main purpose of whose are to verify the protein expression and the interaction between proteins. THP-1 and HUVEC cells are mainly used to study DENV virus infection and changes in endothelial cell permeability. By using different cell lines, we strive to make the experimental results more sufficient and reliable.

There is no discussion on why HUVECs expressing NS1 did not affect MMP-9 expression.

**Authors’ Response:** Good point.

We have detected the level of MMP9 expression in NS1 overexpressed HUVEC cells and found that NS1 had little influence in MMP9 expression. However, NS1 promoted MMP9 expression in a dose-dependent manner in THP-1 cells. We speculated that as the level of MMP9 in HUVEC cells was much lower than that in THP-1 (Revised Fig. 4G), and thus NS1 displayed little effect on MMP9.

In figure 4 the authors evaluate the infection level in THP-1 and HUVECs. They demonstrate increased viral copies in THP-1 and that infection increases with dose of dengue. This experiment needed a UV-inactivated and No RT control to ensure the data is detecting de novo E and not input virus genome.

**Authors’ Response:** Thank you for the very good suggestion.

According to your suggestion, we have performed additional experiments to include the UV-inactivated DENV virus and used the supernatant of C6/36 cells as NO RT control. The new results confirmed that DENV could successfully infect THP-1 cells and HUVECs (Revised Fig. 4A).

In Fig 5. The authors evaluate EC permeability in response to supernatants derived from dengue infected HUVECs or THP-1 cells. There is no indication in the TEER assay that the HUVECs were allowed to establish a tight barrier. The methods do not indicate how many cells were seeded and how long they were left to form barriers. 5J also demonstrates that NS1 alone induced permeability at 7 h post-treatment. This is considerably different than what has been published by both the Harris and King groups. Please explain. And how does the data fit with the lack of MMP-9 generated in HUVECs in response to NS1? What is the factor here that is inducing the permeability to the same levels as NS1 alone? These data do not seem to support the role of MMP-9 as a central mediator but rather simply an additional mechanism that could induce permeability.

**Authors’ Response:** Thank you for the comments.

HUVECs can be used to establish a tight barrier in the TEER assay had been demonstrated previously in our lab (Pan et al. Dengue virus infection activates interleukin-1β to induce tissue injury and vascular leakage. Front Microbiol 2019;10:2637.) and Harris’s lab (Beatty et al., Dengue virus NS1 triggers endothelial permeability and vascular leak that is prevented by NS1 vaccination. Sci Transl Med 2015;7(304):304ra141.). As you suggested, we have supplemented the experimental details in the Methods section in the revised manuscript: HUVECs (2 x 10^5^) were grown on a Transwell insert (0.4 μm; Corning Life Sciences, Corning, NY, USA) until a monolayer formed (about 24 h).

We have demonstrated that NS1 alone could induce changes in the permeability of HUVEC cells (Revised Fig. 5J), which was consistent with previous data reported by Harris’s group. The difference between our results and previous report is that we found that the lowest value appeared at 7 h, while the previous report showed that the lowest value at 9 h. We believed that this difference may be related to the status of cultured cells and the slight difference in the activity of the DENV2-NS1 protein.

As a previous study (Glasner et al., Dengue virus NS1 cytokine-independent vascular leak is dependent on endothelial glycocalyx components. PLOS Pathogens 2017; 13(11):e1006673.) demonstrated that NS1 could induce hyperpermeability by disrupting the endothelial glycocalyx, thus we speculated that MMP9 may be one of the mechanisms underlying the induction of permeability, other mechanism may also involve in such regulation.

In the immunofluorescent experiments (Fig 6) the methods state that HUVECs were seeded onto coverslips and grown to 80% confluency and then treated with NS1, MMP-9 or both for 6 h. Cells were then subsequently stained for tight junction proteins. This is not the correct way to do this experiment. Endothelial cells, including HUVECs, require approximately 4-5 days to establish 100% confluency and develop true tight junctions. This experiment should be repeated with HUVECs that have been allowed to form tight junctions creating a tight barrier.

**Authors’ Response:** Very good question.

According to your suggestion, we have repeated the experiments in the revised manuscript. HUVECs cells were cultured in co-focal plates for 5 days, and then NS1 proteins, MMP9 proteins, and both were added for 6 h. The expression of the junction molecules (β-catenin and ZO-1) was identified by immunofluorescence assay (Revised Fig. 6H and 6I).

In figure 7. The authors use truncated NS1 to determine the interaction domains with tight junction proteins. Given soluble NS1 is a hexamer, and in the cell NS1 exists as a dimer, the authors need to determine how the truncations affect the structure, as this may count for the inability to interact. The data are slightly confusing as to which form of NS1 is mediating what interaction. For the transfection experiments and MMP-9 interaction, this would be dimeric NS1 interacting in the cell. For the modification of tight junction proteins with the addition of NS1 externally, this would suggest the hexamer is mediating the effect. How are the domains that are indicated modeled on the hexamer? Are the on the outside available for interaction or is the interaction abrogated simply because the structure is destroyed?

**Authors’ Response:** Thank you for the comments.

We had performed experiments in the revised manuscript to determine the binding domains of NS1 with MMP9 and β-catenin/ZO-1 through the construction of different truncated mutants of NS1, in order to prove that NS1 plays a bridge role in the interaction between MMP9 and β-catenin/ZO-1 (Original Fig 7I-K, now Revised Fig. S6 A-C). For this purpose, the influence of truncated mutation on the structure of NS1 is ignored. According to your suggestion, we have constructed three new truncated mutants in accordance with the structural domain of NS1. Similar to the previous results, MMP9 could interact with different structural domains of NS1, but β-catenin/zo-1 only interacts with the C-terminal β-ladder domain of NS1 (Revised Fig. S6 D-F). A newly published paper (Modhiran et al., A broadly protective antibody that targets the flavivirus NS1 protein. Science. 2021. PMID: 33414219) appears to explain your question. NS1 interacts with β-catenin/ZO-1 through C-terminal β-ladder domain, demonstrating that β-catenin/ZO-1 could bind extracellular NS1 hexamers. Thus, outside available for interaction between NS1 with β-catenin/ZO-1 was abrogated simply because the structure was destroyed.

**Part III – Minor Issues: Editorial and Data Presentation Modifications**

Please use this section for editorial suggestions as well as relatively minor modifications of existing data that would enhance clarity.

**Reviewer #2:** Fig 1E, F: The TIMP-1 correlations are not very compelling

**Authors’ Response:** Thank you for the comment.

We agree that although the effect of TIMP-1 was proved by experiments in our study, the results include Fig. 1E and F were not very compelling. Therefore, according to your comment and other reviewers, we have removed the results of TIMP-1 in the revised manuscript.

Fig 2: It is unclear how the experiment in Fig 2B differs from the one in Fig 2A.

**Authors’ Response:** Thank you for the comment.

They were reciprocal Co-IP experiments. Fig. 2A demonstrated that DENV NS1 interacts with MMP9, while Fig. 2B confirmed that MMP9 interacts with NS1.

Fig 2D: This co-localization does not seem very robust.

**Authors’ Response:** Good comment.

We agree that the co-localization in Fig. 2D does not seem very robust, thus we have removed this result in the revised manuscript. According to the comments of other reviewer, we have performed additional experiment to show the co-localization in the revised manuscript. HEK293T cells (Revised Fig. 7H) and Hela cells (Revised Fig. 7I) were co-transfected with HA-MMP-9, GFP/HA-MMP-9, GFP-NS1/HA-MMP-9, Flag-β-catenin, Flag-β-catenin/GFP-NS1/HA-MMP-9, Flag-ZO-1, or Flag-ZO-1/GFP-NS1/ HA-MMP-9. The new Immunofluorescence results indicated that NS1 and MMP9 could colocalize at cell-cell junctions (β-catenin/ZO-1) (Revised Fig. 7H and 7I).

Are the NS1 blots in Fig3F and 3G correct?

**Authors’ Response:** Good question.

We have carefully examined the NS1 blots in Fig. 3F and Fig. 3G, and confirmed that these blots are correct.

Fig 4E: The revisions do not address the original question: how is there a single mock for 2 different cell types, with the same number of replicates as the infected samples? Either this is from THP-1 or from HUVEC, but it is not indicated. It doesn’t really matter (this mock doesn’t even need to be included in the figure), but the current presentation doesn’t make sense.

**Authors’ Response:** Thank you very much for the comment.

We are sorry for the miss understand your original suggestion. According to your suggestions, we have corrected the image in the revised manuscript (Revised Fig. 4E).

Fig 5J: The data do not support the conclusion that adding NS1 increased endothelial permeability compared to adding MMP9 alone.

**Authors’ Response:** Good question.

We have compared the TEER value of NS1+MMP9 (mean=0.769) and MMP9 alone (mean=0.822), made a significance analysis of the data, and found that its P value was less than 0.05, indicating a significant difference. Therefore, we believe that this data supports the conclusion. However, we have described it as: NS1 to some extent increased endothelial permeability compared to MMP9 alone.

Fig 7: Check sample designations in 7E and F.

**Authors’ Response:** Thanks.

We have carefully examined the original images of Fig. 7E and found that the blots were correct.

The interaction phenotypes of the truncation mutants don’t make a lot of sense. It also seems that these truncations were not designed with particular consideration of known NS1 domains.

**Authors’ Response:** Good comment.

According to your suggestion, we have constructed three new truncated mutants of NS1 in accordance with the structural domain of NS1 in the revised manuscript. The new results indicated that similar to the previous results, MMP9 could interact with different structural domains of NS1(Domain 1 of NS1 was not detected here because amino acids were too small, so it did not precipitate with MMP9), but β-catenin/zo-1 only interacts with the C-terminal β-ladder domain of NS1 (Revised Fig. S6 D-F).

**Reviewer #3:** 1. Overall, it seems as if MMP9-mediated intercellular junction disruption can occur independently from NS1, which is inconsistent with the model proposed by the authors (that NS1 links the interaction between TJ/AJ proteins and MMP9). How can the authors explain this?

**Authors’ Response:** Thank you for the question.

It is for sure that MMP9 can disrupt the permeability of endothelial cells. But under normal conditions, low level of MMP9 in endothelial cells may not perform this damage. However, upon DENV infection, secreted NS1 recruits MMP9 from immune cells to the surface of endothelial cells to perform this destructive function. This is what we are trying to do that with this model.

2. The authors should describe in further detail the proposed mechanisms previously reported for MMP9 in NS1-mediated pathogenesis (e.g., Chen et al., 2019), as this pathway has been studied previously. It would be helpful to contrast this with the proposed mechanism from this study.

**Authors’ Response:** Good question.

As you suggested, we have citated this article (Chen et al., 2019) and supplemented the discussion. Chen et al found that NS1 protein of ZIKV could promote K63-linked polyubiquitination of MMP9 and stabilize the expression of MMP9, leading to the destruction of the blood-testis barrier. Our results showed that NS1 protein of DENV could promote the expression and activation of MMP9 by activating the NF-κB signaling pathway and directly interacting with MMP9, thus degrading the junction molecules of endothelial cells and thereby promoting the occurrence of vascular leakage. These results demonstrated that MMP9 plays an important role in the pathogenesis of flavivirus infection.

3. The authors need to add scale bars to all IFA images.

**Authors’ Response:** Thank you for the good suggestion.

According to your suggestion, we have supplemented the scale bars to all IFA images in the revised manuscript.

4. In Fig. S4C, the panels for NS1 and MMP9 appear to be the same panels with different signal intensity (these are duplicated panels), indicating a problem with figure design.

**Authors’ Response:** Very good suggestion.

As you suggested, we have revised the panels with same signal intensity in the revised manuscript (Revised Fig. S4C).

5. Lines 302 and 303: this is not the first study to reveal TJ/AJ disruption, please include ([https://doi.org/10.1099/jgv.0.001163](https://doi.org/10.1099/jgv.0.001163)) and (<https://doi.org/10.1099/jgv.0.001401>)

**Authors’ Response:** Thank you very much for the suggestion.

According to the question, we have added the two references (Barbachano-Guerrero et al., Dengue virus non-structural protein 1 activates the p38 MAPK pathway to decrease barrier integrity in primary human endothelial cells. J Gen Virol 2020;101(5):484-496; Singh et al., Dengue virus or NS1 protein induces trans-endothelial cell permeability associated with VE-cadherin and RhoA phosphorylation in HMEC-1 cells preventable by angiopoietin-1. J Gen Virol 2018;99(12):1658-1670.) in the revised manuscript (Revised line 322).

6. Line 142: “opt” should be “cooperate”.

**Authors’ Response:** Thank you.

We have corrected the “opt” to “cooperate” in the revised manuscript.

7. Line 203, do authors mean NS1-induced MMP9 expression?

**Authors’ Response:** Thanks again.

Yes, we did mean NS1-induced MMP9 expression in Line 209. We have corrected this description in the revised manuscript.

8. Lines 310-311, this statement is not justified by the data.

**Authors’ Response:** Thank you for the comment.

According to our results (Revised Fig. 6 A-F), we think that the statement is justified by the data in the revised manuscript (Revised line 310-311).
