# Supplementary material for: DENV NS1 and MMP-9 cooperate to induce vascular leakage by altering endothelial cell adhesion and tight junction
Source: PLoS Pathog. 2021 Jul 26;17(7):e1008603. doi: 10.1371/journal.ppat.1008603 (PMC8341711; doi:10.1371/journal.ppat.1008603)
Supplement: Attachment — Submitted filename: PLOS Pathog Responses to the Reviews Pan-Wu -20210429.doc [file ppat.1008603.s015.doc]

**Responses to the Reviews’ Comments**

**Part I – Summary**

Please use this section to discuss strengths/weaknesses of study, novelty/significance, general execution and scholarship.

**Reviewer #2:** The authors have added additional data and responded to reviewer comments.

**Authors’ Response:** Thank you very much for the Summary.

**Reviewer #3:** This revised manuscript by Pan et al., is improved but still lacks key experiments to support the authors central hypotheses of how an interaction between NS1 and MMP9 alters endothelial dysfunction. As stated in the previous review, a role for MMP9 in NS1-mediated endothelial dysfunction has been previously reported, and the advancement of this paper is that a direct protein-protein interaction between NS1 and MMP9 (1) modulates enzymatic activity of MMP9 and (2) localizes secreted MMP9 to the TJ/AJ of endothelial cells. While these conclusions of the authors are certainly reasonable, they are not yet fully supported by the data. The authors must address the following key points detailed below.

**Authors’ Response:** Thank you for your comments.

According to your suggestion, we have performed additional experiments to support that a direct protein-protein interaction between NS1 and MMP9 modulates enzymatic activity of MMP9 and localizes secreted MMP9 to the TJ/AJ of endothelial cells in the revised manuscript. Please see Part II and III for details.

**Reviewer #4:** The authors have addressed many of the concerns I had.

**Authors’ Response:** Thank you very much for your Summary.

**Part II – Major Issues: Key Experiments Required for Acceptance**
Please use this section to detail the key new experiments or modifications of existing experiments that should be absolutely required to validate study conclusions.

Generally, there should be no more than 3 such required experiments or major modifications for a "Major Revision" recommendation. If more than 3 experiments are necessary to validate the study conclusions, then you are encouraged to recommend "Reject".

**Reviewer #2: (No Response)**

**Reviewer #3:** 1. While it is clear that a direct interaction between NS1 and MMP9 is occurring, the authors have not yet established that this is not simply a non-specific interaction owing to stickiness of MMP9 (specifically AA 440-707 according to their MMP9 truncation mutants). To test this, the authors require a proper negative control in their CO-IP, BiFC, and Y2H assays where a protein not interacting with MMP9 is successfully expressed/pulled down, and shown NOT to interact with MMP9; simply an empty vector where no protein is expressed is not an adequate control. The authors could take advantage of one of their DENV expression constructs, such as E, or any other nonstructural proteins, as tested in Fig. 2A, which appears to be in hand and would serve the purpose.

**Authors’ Response:** Thank you for the good suggestion.

As you suggested, we have performed additional experiments to demonstrate the specific interaction between NS1 and MMP9 by using DENV structural protein E and nonstructural protein NS4A as controls in the revised manuscript. New results from BIFC assays demonstrated that NS1 interacted with MMP-9, but E and NS4A could not interact with MMP-9, in both HEK293T cells (Revised Fig 2C and D) and Hela cells (Revised S1A and B Fig). In addition, yeast-two hybridization assays confirmed that NS1 and MMP-9 could interact with each other (Revised Fig 2F), but E or NS4A failed to interact with MMP-9 (Revised S1C Fig).

2. The authors need to address the function of this interaction, for example by: (1) repeating the gelatinase assay with 3 separate conditions using NS1, NS3, and E (these conditions would address whether gelatinase enhancement of MMP9 is specific to NS1, protein-protein interactions, or if it is non-specific); (2) making mutants of NS1 or MMP9 that no longer interact and demonstrate the lack of function with these proteins.

**Authors’ Response:** Thank you for the suggestions.

According to your suggestions, we have carried out additional experiments in the revised manuscript.

The new data revealed that in transfected PMA-differentiated THP-1 cells, E and NS3 had no effect on MMP-9 enzyme activity or MMP-9 protein production (Revised S2I and J Fig) and that unlike NS1, NS3 and E did not play roles in the regulation of MMP-9 enzyme activity and MMP-9 protein production (Revised S2K Fig), demonstrating that gelatinase enhancement and protein production of MMP9 are specific to NS3, but not to E and NS3.

New results from BIFC assays demonstrated that NS1 interacted with MMP-9, but E and NS4A could not interact with MMP-9, in both HEK293T cells (Revised Fig 2C and D) and Hela cells (Revised S1A and B Fig), and yeast-two hybridization assays confirmed that NS1 and MMP-9 could interact with each other (Revised Fig 2F) but E or NS4A failed to interact with MMP-9 (Revised S1C Fig), confirming that NS1-MMP-9 protein-protein interaction is specific.

The domains of MMP-9 involved in the MMP-9/NS1 interaction were determined by progressive truncation of MMP-9 (D1–D9) (Revised Fig 2G). HEK293T cells were co-transfected with pHA-NS1 along with each of the plasmids expressing truncated MMP-9 protein, respectively. Co-IP results showed NS1 interacted with MMP-9 D1 (106–707), MMP-9 D4 (106–511), MMP-9 D5 (1–511), MMP-9 D6 (106–440), MMP-9 D7 (106–233), MMP-9 D8 (106–397), and MMP-9 D9 (223–440), but not with MMP-9 D2 (440–707) or MMP-9 D3 (512–707), suggesting that the Zinc-binding catalytic domain and the Fibronectin type-like domain D6 (106–440) are involved in the MMP-9/NS1 interaction (Revised Fig 2H).

As the Zinc-binding catalytic domain and the Fibronectin type-like domain D6 (106–440) are involved in the MMP-9/NS1 interaction and essential for MMP-9 function, and thus, we cannot use the mutants to address your comment: making mutants of NS1 or MMP9 that no longer interact and demonstrate the lack of function with these proteins.

Moreover, the binding sites of NS1 involved in the NS1/MMP-9, NS1/β-catenin, NS1/ZO-1 interaction were determined by progressive truncation of NS1 (NS1-1–NS1-8) (Revised S7A Fig). HEK293T cells were co-transfected with pHA-MMP-9 along with each of the plasmids expressing truncated NS1 protein, respectively. Co-IP results showed MMP-9 interacted with all the domains of NS1 (Revised S7B Fig). HEK293T cells were transfected with each of the plasmids expressing truncated NS1 protein, respectively. Co-IP results showed that endogenous β-catenin interacted with NS1-1 (1–292), NS1-5 (72–352), NS1-6 (142–352), NS1-7 (212–352), and NS1-8 (282–352), but not with NS1-2 (1–212), NS1-3 (1–142), or NS1-4 (1–72) (Revised S7C Fig), suggesting that the domain NS1-7 (212–352) are involved in NS1/β-catenin interaction. The results also indicated that endogenous ZO-1 interacted with NS1-5 (72–352), NS1-6 (142–352), NS1-7 (212–352), and NS1-8 (282–352), but not with NS1-1 (1–292), NS1-2 (1–212), NS1-3 (1–142), or NS1-4 (1–72) (Revised S7C Fig), suggesting that the domain NS1-8 (282–352) are involved in NS1/ZO-1 interaction. We next constructed three new truncated mutants in accordance with the structural domain of NS1 (Revised S7D Fig). Similar to the previous results, MMP9 could interact with different structural domains of NS1 (Domain 1 of NS1 was not detected here because amino acids were too small to be precipitated with MMP9) (Revised S7E Fig), but β-catenin/ZO-1 only interacts with the C-terminal β-ladder domain of NS1 (Revised S7F Fig).

As MMP-9 interacted with all the domains of NS1, and thus again we cannot use NS1 mutants to address your comment: making mutants of NS1 or MMP9 that no longer interact and demonstrate the lack of function with these proteins.

3. Regarding the gelatinase assay in Figure 3H, could the authors explain why the gelatinase activity of MMP9 increases in the presence of NS1 even though pro-MMP9 does not seem to mature to a lower band on the Western blot? How do the authors propose that NS1 increases MMP9 enzymatic activity? It doesn’t seem to be promoting maturation of pro-MMP9. Could the authors also display more of this Western blot, it appears that a lower band may be cut off?

**Authors’ Response:** Again, thank you for your comments.

According to your suggestion, we have repeated the experiments in the revised manuscript. The new data showed that MMPs are secreted as inactive proenzymes and are activated subsequently by the cleavage of the propeptide domain by proteolytic enzymes. Results from gelatinase activity assays showed that gelatinase activity was only detected in the presence of both NS1 protein and pro-MMP9 protein, but the enzyme activity was not detected in the presence of BSA protein, NS1 protein, or pro-MMP9 protein alone, or in the presence of BSA and pro-MMP9 (Revised Fig 3H). The new results demonstrate that NS1 regulates the protease activity of MMP9 by interacting with MMP9 to promote the maturation of pro-MMP-9.

4. Finally, since the authors claim that NS1 and MMP9 localize to the TJ/AJ of cells, this needs to be shown. Overexpressing TJ/AJ proteins in HeLa and 293T cells is not sufficient to demonstrate this point, as these proteins are clearly localized everywhere in the cytoplasm and are not forming actual junctional complexes. The best way to do this colocalization would be using a relevant model of polarized human endothelial cells such as HUVECs and detect endogenous TJ/AJ proteins (many good antibodies exist) and determine whether extracellular NS1 and MMP9 colocalize. If this could not be done, the authors would need to revise their overall model to exclude the steps not supported by data.

**Authors’ Response:** Thanks again.

We noticed that over-expression of TJ/AJ proteins in HeLa and 293T cells was not sufficient to demonstrate that NS1 and MMP9 co-localize to the TJ/AJ of cells. Therefore, we have removed the previous results (Original Fig. 7H and I) from the revised manuscript.

According to your suggestion, we have performed additional experiments by using and analyzing HUVEC cells treated with active NS1 protein and MMP-9 protein. HUVECs were incubated with commercialized DENV2 NS1 protein and recombinant human MMP-9 protein. Immunofluorescence results revealed that extracellular NS1 protein and MMP-9 protein were co-localized with each other, and they were also co-located with β-catenin (Revised Fig 7K) and ZO-1 (Revised Fig 7L). We also noticed that the structures of β-catenin and ZO-1 were destroyed in the presence of NS1 and MMP-9 in HUVEC cells (Revised Fig 7K, L). There results confirm that NS1 recruits MMP-9 to disrupt the junctions between endothelial cells.

5. Finally, all IFA should be quantified in some way, especially the colocalization data (BiFC and colocalization figures), as it is important to demonstrate that the images are truly representative of the overall trend.

**Authors’ Response:** Thank you for the suggestion.

According to your suggestion, all IFA have been quantified by using ImageJ software in the revised manuscript (Revised Fig. 2C and D; Revised Fig. 6H–K; Revised Fig. 7K and L, right; Revised Fig. S1A and B; Revised Fig. S5B–E).

**Reviewer #4: (No Response)**

**Part III – Minor Issues: Editorial and Data Presentation Modifications**

Please use this section for editorial suggestions as well as relatively minor modifications of existing data that would enhance clarity.

**Reviewer #2:** Line 155-156: A correlation between NS1 and MMP9 levels in serum does not suggest anything about a mechanistic role for an interaction between the two, please rephrase or delete.

**Authors’ Response:** Thank you for the good suggestion.

According to your suggestion, we have changed the description “These results suggest that the NS1 and MMP-9 interaction may play an important role in severe dengue patients.” to “These results suggest that MMP9 production is enhanced in severe dengue patients and is correlated with NS1 production.” in the revised manuscript.

Line 197-199: It is unclear how low MMP9 expression in HUVEC is an explanation for the inability of NS1 to induce its expression. Fig 4G is called out of order in the text.

**Authors’ Response:** Thank you for the comment.

We have modified the description and deleted original Fig 4G in the revised manuscript based on your suggestion. “MMP-9 protein production and MMP-9 enzyme activity were facilitated by NS1 in dose-dependent manners in HEK293T cells (Revised Fig 3B). We also noticed that level of MMP-9 protein was induced by NS1 in THP-1 differentiated macrophages (Revised S2G Fig) but relatively unaffected by NS1 protein in HUVECs (Revised S2H Fig). We speculated that one of the reasons for this phenomenal is the expression level of MMP-9 in HUVECs was much lower than that in THP-1 (Revised S2G and H Fig).”.

Fig 9: change “adherent junction” to “adherens junction”

**Authors’ Response:** Thank you for the suggestion.

We have changed “adherent junction” to “adherens junction” in the revised manuscript (Revised Fig 9) according to your suggestion.

**Reviewer #3:** Could the authors add more detail to their Y2H protocol? Is the Figure correct with the dropout media combination (-TRP,-LEU,-HIS,-ADE), or is this the negative control condition? What about the -TRP,-LEU,-HIS condition? Clarifying the complementation combination and yeast strain would clarify this point.

**Authors’ Response:** Thank you for the comments.

According to your suggestion, we have added more detail to the protocol of Y2H in the Materials and Methods Section in the revised manuscript.

Human MMP-9 gene was subcloned in pGBKT7, and DENV NS1, E, and NS4A were subcloned in pGADT7 respectively. control vectors pGBKT7, pGADT7, pGBKT7-p53, pGADT7-T, and pGBKT7-lam, and some reagents were purchased from Clontech Laboratories. All experimental procedures were done following the Matchmaker Gold Yeast 2-Hybrid System User Manual. Briefly, yeast strain AH109 cells were co-transformed with plasmid pGADT7 and plasmid pGBKT7. Transformed yeast cells were grown in SD-minus Trp/Leu plates (DDO) for 24–48 h, and then subcloned replica plated on SD-minus Trp/Leu/Ade/His plate (QDO) for another 48–96 h.

The SD/-Trp-Leu means the dropout media combination (-Trp and -Leu) and SD/-Trp-Leu -His-Ade means the dropout media combination (-TRP, -LEU, -HIS, -ADE) (Revised Fig 2F and Revised S1C Fig). We did not use the dropout media combination (-TRP, -LEU, -HIS) in our study.

**Reviewer #4: Minor points.**

The authors stated that the new fig 1 had each individual patient as a different symbol as requested. This is not apparent on the figure.

**Authors’ Response:** Thank you for the comment.

Sorry for the mistake. Now, we have added individual patient as a different symbol in the revised manuscript (Revised Fig 1A–D).

Lines 186-190 do not make sense

**Authors’ Response:** Thanks again for your comment.

In the original Lines 186-190, we simply wanted to describe the results of cell transfections, which showed that the proteins were produced in transfected cells. To make it clearer, we have corrected the description in the revised manuscript.

The ELISA results indicated that the levels of NS1 protein in the supernatants were increased in pHA-NS1-concentration dependent fashions (Revised S2A–D Fig) and similarly, Western-blot results showed that the levels of NS1 protein in the cell lysates were increased in pHA-NS1 concentration-dependent manners (Revised S2E–H Fig), demonstrating that NS1 protein is secreted in the supernatants and expressed in the cell of these cell lines.

In lines 251-253 the authors indicate that THP-1 cells induce high levels of MMP9 in response to dengue infection while HUVECs exhibited ‘modest’ elevation in MMP9. Looking at the presented data in Fig 4G there is no production of MMP9. The statement is not correct.

**Authors’ Response:** Thank you for the comment.

We have modified the description and deleted original Fig 4G in the revised manuscript based on your suggestion. “MMP-9 protein production and MMP-9 enzyme activity were facilitated by NS1 in dose-dependent manners in HEK293T cells (Revised Fig 3B). We also noticed that level of MMP-9 protein was induced by NS1 in THP-1 differentiated macrophages (Revised S2G Fig) but relatively unaffected by NS1 protein in HUVECs (Revised S2H Fig). We speculated that one of the reasons for this phenomenal is the expression level of MMP-9 in HUVECs was much lower than that in THP-1 (Revised S2G and H Fig).”.

Line 214. Capitalize Results

**Authors’ Response:** Thank you for the suggestion.

According to your suggestion, we have change “results” to “Results” in the revised manuscript.

We thank all reviewers for their constructive and insightful comments, which have helped us substantially improve our manuscript.
