# Supplementary material for: DENV NS1 and MMP-9 cooperate to induce vascular leakage by altering endothelial cell adhesion and tight junction
Source: PLoS Pathog. 2021 Jul 26;17(7):e1008603. doi: 10.1371/journal.ppat.1008603 (PMC8341711; doi:10.1371/journal.ppat.1008603)
Supplement: Attachment — Submitted filename: PLOS Pathog Responses to the Reviews Pan-Wu -20210701.docx [file ppat.1008603.s016.docx]

**Responses to the Reviews’ Comments**

**Reviewer's Responses to Questions**

**Part I – Summary**

Please use this section to discuss strengths/weaknesses of study, novelty/significance, general execution and scholarship.

**Reviewer #3:** The authors have addressed some of my concerns, but issues remain. The authors have now sufficiently demonstrated that NS1 interacts specifically with MMP9 (although it is strange that all domains of NS1 interact with MMP9, suggesting that NS1 is a sticky protein). However, while the authors provide some evidence that NS1 promotes maturation of MMP9 and increased gelatinase activity, the functional relevance of this interaction remains unclear given the current data. Further, the subcellular localization and colocalization of NS1/MMP9 remain unclear, which is critical given the model drawn by the authors.

**Authors’ Response:** Thank you for the comments.

According to your suggestion, we have added the description of “However, the detailed functional relevance of the interaction between NS1 and MMP-9 needs further investigations in the future.” in the discussion section of the revised manuscript.

In addition, we have performed additional experiments to determine the colocation of NS1 and MMP-9. HUVECs were respectively incubated with commercialized DENV2 NS1 protein, recombinant human MMP-9 protein, and NS1 plus MMP-9 protein. The new immunofluorescence results revealed that when presented individually, NS1 protein (Revised Fig 7Ka–e) or MMP-9 protein (Revised Fig 7Kf–j) was distributed outside the nucleus; when precented together, extracellular NS1 protein and MMP-9 protein were co-localized with each other (Revised Fig 7Kk–p) and distributed in cell membrane (Revised Fig 7Kq–v). Notably, NS1 and MMP-9 protein also co-located with β-catenin (Revised Fig 7Kac–ah) and ZO-1 (Revised Fig 7Kau–az). We also noticed that compared with untreated cells (Revised Fig 7Kw–ab and Revised 7Kai–an), the structures of β-catenin (Revised Fig 7Kac–ah) and ZO-1 (Revised Fig 7Kau–ay) were destroyed in the presence of NS1 and MMP-9 in HUVEC cells (Revised Fig 7K, L). There results confirm that NS1 recruits MMP-9 to disrupt the junctions between endothelial cells.

**Reviewer #4:** The authors have addressed my concerns.

**Authors’ Response:** Thank you very much.

**Part II – Major Issues: Key Experiments Required for Acceptance**

Please use this section to detail the key new experiments or modifications of existing experiments that should be absolutely required to validate study conclusions.

Generally, there should be no more than 3 such required experiments or major modifications for a "Major Revision" recommendation. If more than 3 experiments are necessary to validate the study conclusions, then you are encouraged to recommend "Reject".

**Reviewer #3:** 1. The authors should discuss the caveats of their data, stating clearly that the functional relevance of the interaction between NS1 and MMP9 is not demonstrated in this paper.

**Authors’ Response:** Thank you again for the comment.

Again, according to your suggestion, we have added the description of “However, the detailed functional relevance of the interaction between NS1 and MMP-9 needs further investigations in the future.” in the discussion section of the revised manuscript.

2. The IFA in Figure 7K and L still lack controls: they require treatment of MMP9 and NS1 alone as well as in combination. Further, Fig. 7K does not show colocalization of MMP9 and NS1; the green and red stainings in the perinuclear region are clearly separate and not overlapping. Representative images need to show multiple, confluent cells (1) to demonstrate reakdown of intercellular junctions and (2) to demonstrate that co-localization occurs and is representative of the entire sample. Lastly, the localization appears to be intracellular (perinuclear), which is inconsistent with the author’s overall model. The NS1 staining pattern seems to be atypical of what is observed by others as well.

**Authors’ Response:** Thank you for your comments.

According to your suggestion, we have performed additional experiments to determine the colocation of NS1 and MMP-9. HUVECs were respectively incubated with commercialized DENV2 NS1 protein, recombinant human MMP-9 protein, and NS1 plus MMP-9 protein. The new immunofluorescence results revealed that when presented individually, NS1 protein (Revised Fig 7Ka–e) or MMP-9 protein (Revised Fig 7Kf–j) was distributed outside the nucleus; when precented together, extracellular NS1 protein and MMP-9 protein were co-localized with each other (Revised Fig 7Kk–p) and distributed in cell membrane (Revised Fig 7Kq–v). Notably, NS1 and MMP-9 protein also co-located with β-catenin (Revised Fig 7Kac–ah) and ZO-1 (Revised Fig 7Kau–az). We also noticed that compared with untreated cells (Revised Fig 7Kw–ab and Revised 7Kai–an), the structures of β-catenin (Revised Fig 7Kac–ah) and ZO-1 (Revised Fig 7Kau–ay) were destroyed in the presence of NS1 and MMP-9 in HUVEC cells (Revised Fig 7K, L). There results confirm that NS1 recruits MMP-9 to disrupt the junctions between endothelial cells.

**Reviewer #4: (No Response)**

**Authors’ Response**: Many thanks.

**Part III – Minor Issues: Editorial and Data Presentation Modifications**

Please use this section for editorial suggestions as well as relatively minor modifications of existing data that would enhance clarity.

**Reviewer #3**: The authors ideally should provide controls demonstrating expression of E and NS3 for their new figures (Figure 2C-D, Figure S1A-C and F).

**Authors’ Response:** Thank you for your comments.

As you suggested, we have determined the expression statuses of the related proteins by Western-blot analyses in the revised manuscript. The new results showed that MMP-9, NS1, E, and NS4A proteins were expressed and detected in both HEK293T cells (Revised S1A Fig) and Hela cells (Revised S1B Fig). Moreover, new Western-blot results confirmed that MMP-9, NS1, E, and NS4A proteins were expressed and detected in the yeast cells (Revised S1F Fig).

**Reviewer #4: (No Response)**

**Authors’ Response:** Thanks again.
